# Unusual reaction of (*E*)-2-[(benzo[*d*]thia­zol-2-yl­imino)­meth­yl]-5-(di­ethyl­amino)­phenol with tri­phenyl­borane: crystal structures and optical properties

**DOI:** 10.1107/S2056989023008514

**Published:** 2023-10-03

**Authors:** Hai Le Thi Hong, Thao Le Phuong, Thong Van Pham, Hue Minh Thi Nguyen, Luc Van Meervelt

**Affiliations:** a Department of Chemistry, Hanoi National University of Education, 136 Xuan Thuy, Cau Giay, Hanoi, Vietnam; b Department of Chemistry, KU Leuven, Biomolecular Architecture, Celestijnenlaan 200F, Leuven (Heverlee), B-3001, Belgium; University of Kentucky, USA

**Keywords:** crystal structure, benzo­thia­zole, boranils, fluorescence, aggregation-induced emission

## Abstract

The mol­ecular and crystal structure of (*E*)-2-[(benzo[*d*]thia­zol-2-yl­imino)­meth­yl]-5-(di­ethyl­amino)­phenol (C_18_H_19_N_3_O_2_S, **Et_2_N-Bz**) and its unexpected reaction product with tri­phenyl­borane, 2,2-diphenyl-1,3-dioxa-2-borata-1,2-di­hydro­naphthalene [systematic name: *N*,*N*-diethyl-2,2-diphenyl-2*H*-1,3λ^3^,2λ^4^-ben­zodioxaborinin-7-amine, C_23_H_24_BNO_2_, (**I**)] are described. For **Et_2_N-Bz**, the hydroxyl group is involved in an intra­molecular hydrogen bond with the imino nitro­gen atom and the C=N bond displays an *E* configuration. The crystal packing is characterized by layers of inversion dimers parallel to the (10  ) plane and chains of mol­ecule in the *a*-axis direction formed through C—H⋯O inter­actions. Complex (**I**) crystallizes with two mol­ecules (*A* and *B*) in the asymmetric unit, which differ in the orientation of the ethyl groups. The 1,3-dioxa-2-borata-1,2,3,4-tetra­hydro­naphthalene ring displays a slight envelope conformation with the boron atom as the flap. In the crystal packing, chains of alternating *A* and *B* mol­ecules formed by C—H⋯O hydrogen bonds run in the *b*-axis direction. The UV–vis absorption and emission properties of the compounds are discussed and their aggregation-induced emission properties are further investigated.

## Abstract 2

The mol­ecular and crystal structure of (*E*)-2-[(benzo[*d*]thia­zol-2-yl­imino)­meth­yl]-5-(di­ethyl­amino)­phenol and its reaction product with tri­phenyl­borane are described. In compound **Et_2_N-Bz**, one of the ethyl groups and the benzo­thia­zole ring are disordered over two sets of atomic sites with major occupancy components of 0.822 (5) and 0.843 (2), respectively.

## 1. Chemical context

Recently, boron complexes have gained increasing attention in fluorescent materials because they have many potential applications in the field of photoelectric devices, fluorescent sensors and probes (Li *et al.*, 2013 ▸; Shi *et al.*, 2020 ▸). Among them, boranils, *i.e.* boron complexes using salicylaldimine as a ligand, have emerged as promising materials due to their excellent optical properties, ICT (inter­molecular charge transfer), high Stokes shift and simple synthesis (Vidyasagar *et al.*, 2019 ▸). An additional advantage of boranils is that their emission characteristics can be adjusted in a flexible way through structural changes such as extending the π-conjugation system, adding donor/acceptor substituents, increasing mol­ecular rigidity and flattening the structures (Frath *et al.*, 2014 ▸; Zhao *et al.*, 2019 ▸; Macé *et al.*, 2021 ▸; Al-Sharif *et al.*, 2020 ▸). These complexes can be synthesized on a multi-gram scale with a two-step process, including synthesis of a Schiff-base ligand *via* a condensation reaction between an amine and an hy­droxy­aldehyde, and complexation with commercial boron compounds (Massue *et al.*, 2021 ▸). In addition, Schiff bases containing the benzo­thia­zole component have a wide range of bioapplications (Shinde & Waghamode, 2017 ▸; Ceramella *et al.*, 2022 ▸; Bhat *et al.*, 2017 ▸), but their optical potential does not seem to have received much attention. Recently, several studies have shown that these derivatives can be used as fluorescent chemosensors in living cells (Khan *et al.*, 2021 ▸), aggregation-induced emission (AIE) active materials (Kachwal *et al.*, 2018 ▸) and potential non-linear optical mat­erials (Muhammad *et al.*, 2018 ▸). 
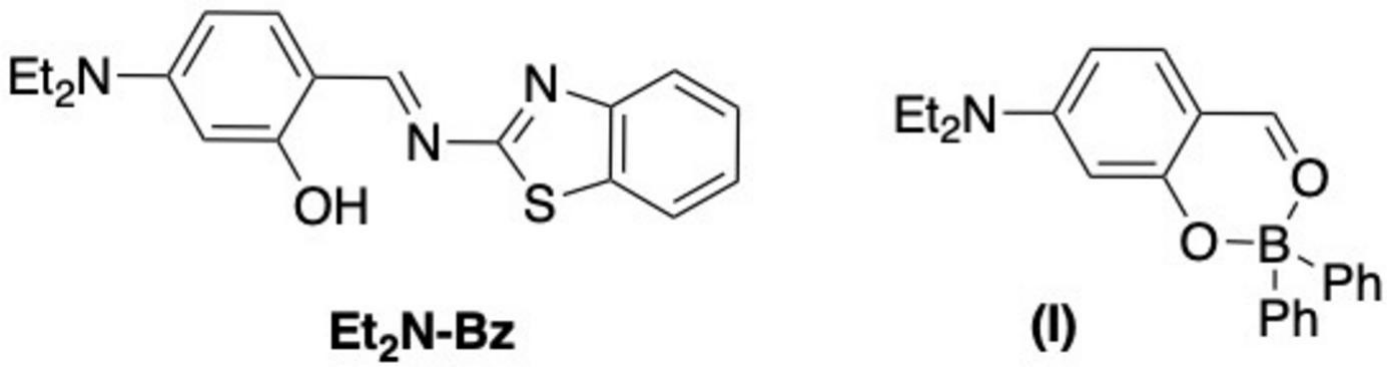


**Figure 1:**
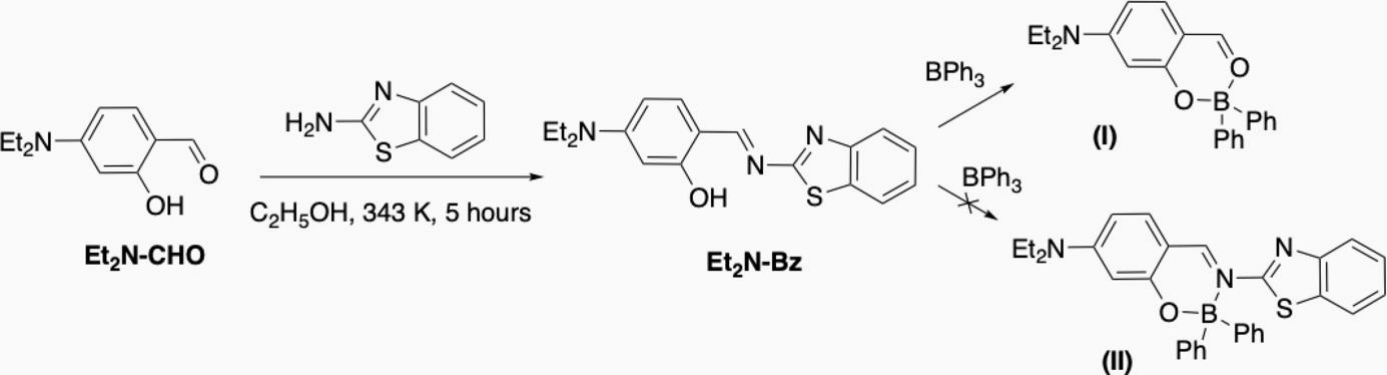
Synthesis of compounds **Et_2_N-Bz** and (**I**).

In this study, we intended to design a new boron(III) complex by replacing the amine component in the structure of boranils with 2-amino­benzo­thia­zole to extend their π-conjugated system. From this idea, (*E*)-2-[(benzo[*d*]thia­zol-2-yl­imino)­meth­yl]-5-(di­ethyl­amino)­phenol (compound **Et_2_N-Bz**) was synthesized with high efficiency *via* a condensation reaction between 2-amino­benzo­thia­zole and 4-(di­ethyl­amino)-2-hy­droxy­benzaldehyde (Fig. 1 ▸). As planned, boron complex (**II**) would be formed by reaction between ligand **Et_2_N-Bz** and BPh_3_ (triphenyl borane). In the expected complex, boron would coordinate with the ligand through the oxygen atom of the hydroxyl group and the nitro­gen atom of the imine group. But more surprisingly, the results of NMR and SC-XRD analysis indicated that the product obtained had structure (**I**) instead of the expected structure (**II**). This phenomenon can be explained by the fact that due to the simultaneous presence of Lewis acid BPh_3_ in the CHCl_3_ solvent, ligand **Et_2_N-Bz** is hydrolyzed and the boron atom is cyclized with the two oxygen atoms. To further elucidate this assumption, the inter­action of 4-(di­ethyl­amino)-2-hy­droxy­benzaldehyde (**Et_2_N-CHO**) with BPh_3_ has been tested under similar experimental conditions. However, the TLC analysis results showed that no compounds were formed. The crystal structures and photophysical properties of the ligand **Et_2_N-Bz** and complex (**I**) are presented in this work.

## 2. Structural commentary

**Figure 2:**
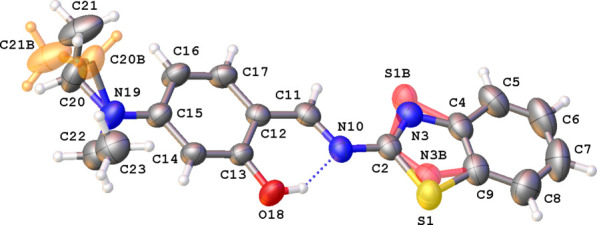
The mol­ecular structure of **Et_2_N-Bz** showing the atom-labeling scheme and displacement ellipsoids at the 30% probability level. The intra­molecular O—H⋯N hydrogen bond is shown as a dashed line. Minor disorder components are shown in orange (ethyl group) and red (benzo­thia­zole ring).

Compound **Et_2_N-Bz** crystallizes in the monoclinic space group *P*2_1_/*n* with one mol­ecule in the asymmetric unit (Fig. 2 ▸). One of the ethyl groups (C20–C21) and the benzo­thia­zole ring are disordered over two sets of atomic sites with major occupancy components of 0.822 (5) and 0.843 (2), respectively. The Schiff base displays an *E* configuration with respect to the C11=N10 double bond. The benzo­thia­zole ring is planar [maximum deviation = 0.010 (3) Å for N3] and subtends a dihedral angle of 5.08 (7)° with phenyl ring C12–C17. The hydroxyl group O18—H18 is involved in an intra­molecular hydrogen bond with the imino nitro­gen atom N10 (Fig. 2 ▸, Table 1 ▸). One of the orientations of the benzo­thia­zole group shows a short intra­molecular contact (H11⋯S1*B* = 2.46 Å).

**Figure 3:**
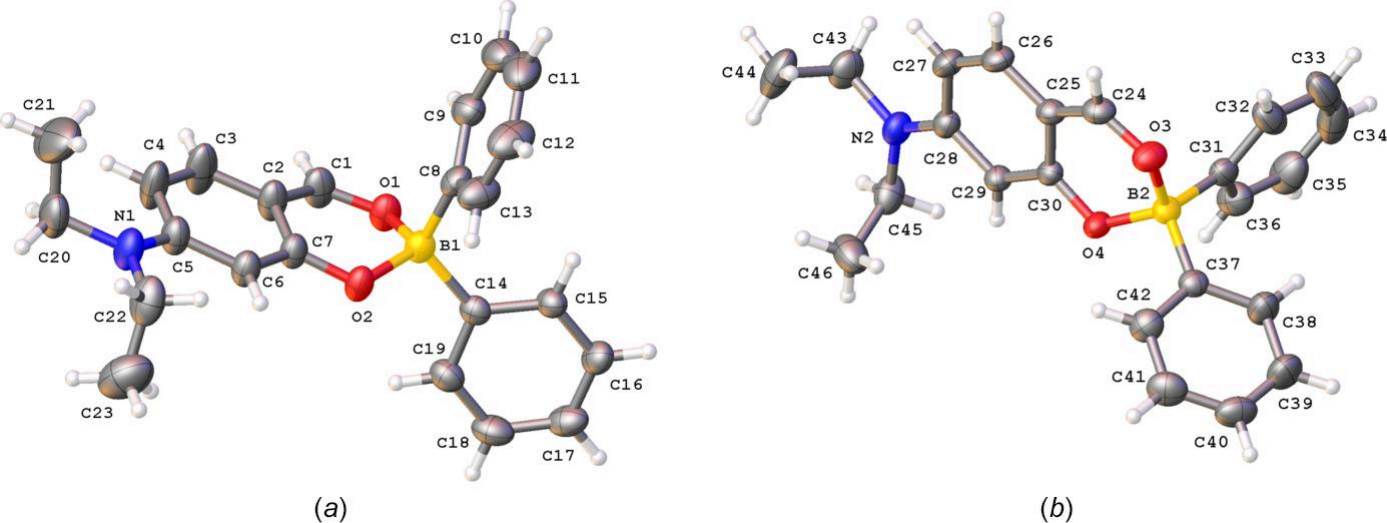
The mol­ecular structure of mol­ecules *A* and *B* in the asymmetric unit of (**I**) showing the atom-labeling scheme and displacement ellipsoids at the 30% probability level.

Complex (**I**) crystallizes in the triclinic space group *P* 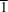 with two mol­ecules in the asymmetric unit (Fig. 3 ▸). The r.m.s. deviation for the best fit (with inversion) of the two mol­ecules is 0.849 Å. The orientations of the ethyl groups differ in mol­ecules *A* (containing atom B1) and *B* (containing atom B2). In mol­ecule *A*, the ethyl groups are on a different side of the ring to which the di­ethyl­amino group is attached, whereas in mol­ecule *B* both ethyl groups are on the same side. The 1,3-dioxa-2-borata-1,2,3,4-tetra­hydro­naphthalene ring shows a slight envelope conformation with the boron atom as the flap. For mol­ecule *A*, the deviation of atom B1 from the best plane through the ring is 0.315 (3) Å, for mol­ecule *B* the deviation for B2 is 0.301 (3) Å. For mol­ecule *A*, this boron-containing plane makes dihedral angles of 84.96 (14) and 81.09 (12)° with phenyl rings C8–C13 and C14–C19, respectively. For mol­ecule *B*, the boron-containing plane makes angles of 77.83 (14) and 81.92 (12)° with phenyl rings C31–C36 and C37–C42, respectively.

## 3. Supra­molecular features

**Figure 4:**
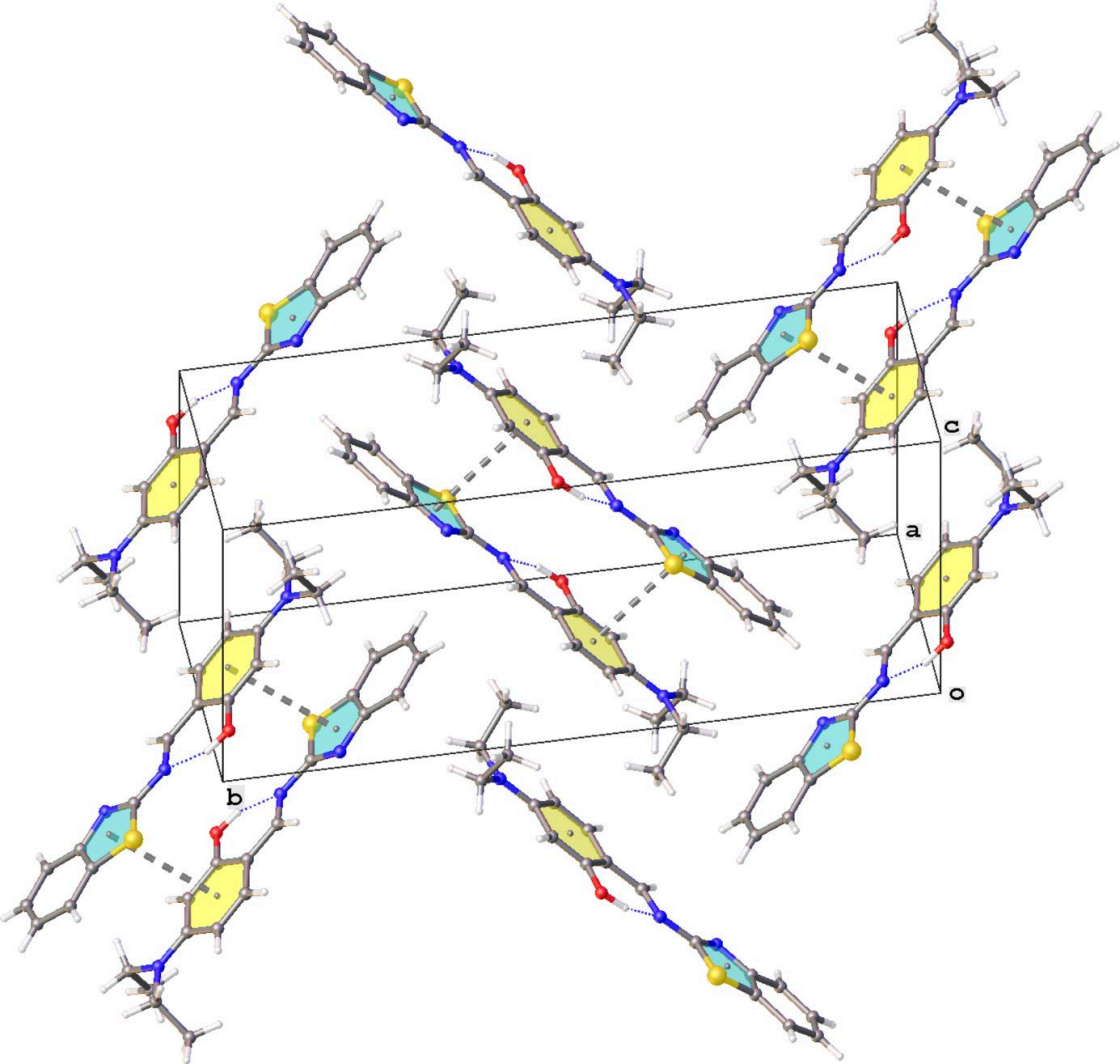
Formation of inversion dimers showing π–π stacking between the phenyl C12–C17 (yellow) and thia­zole S1/C2/N3/C4/C9 (blue) rings in the crystal packing of **Et_2_N-Bz**.

**Figure 5:**
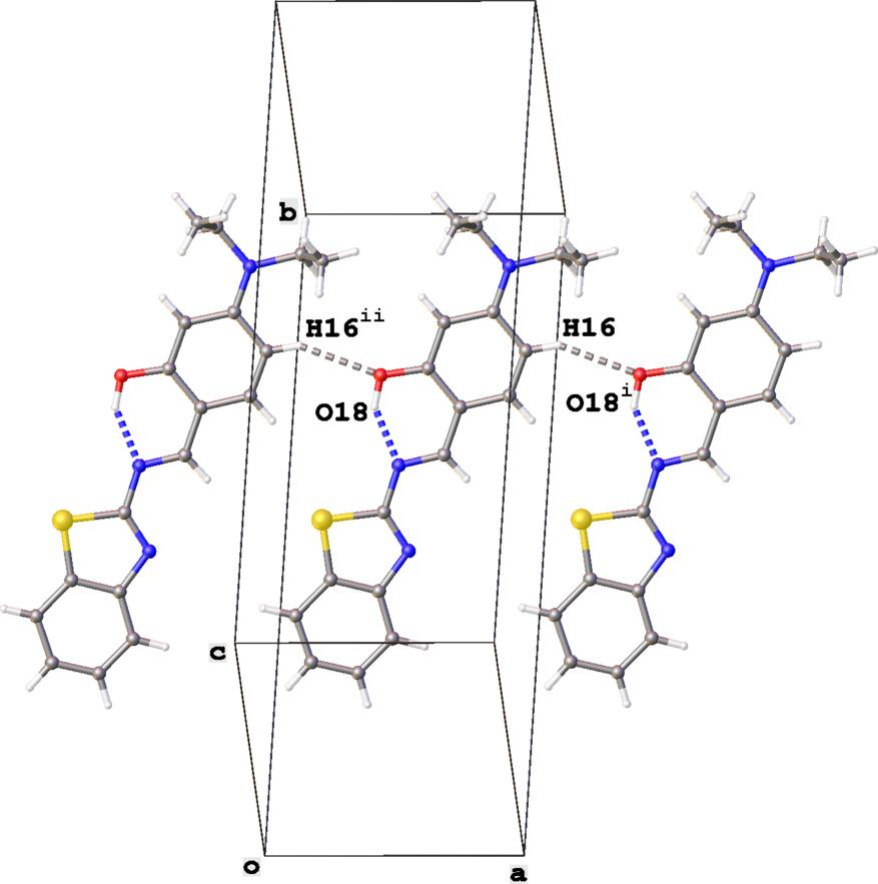
Chain of mol­ecules running in the *a*-axis direction in the crystal packing of **Et_2_N-Bz**. The O—H⋯N and C—H⋯O hydrogen bonds are shown as blue and gray dashed lines, respectively. Only major disorder components are shown. Symmetry codes: (i) 1 + *x*, *y*, *z*; (ii) −1 + *x*, *y*, *z*.

The crystal packing of **Et_2_N-Bz** is characterized by the formation of inversion dimers showing π–π stacking between the phenyl (C12–C17) and thia­zole (S1/C2/N3/C4/C9) rings [centroid–centroid distance = 3.7856 (13) Å]. The dimers form layers parallel to the (10 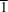 ) plane as shown in Fig. 4 ▸. Neighboring layers inter­act through C16—H16⋯O18^i^ hydrogen bonds that form chains of mol­ecules in the *a*-axis direction (Fig. 5 ▸. see Table 1 ▸ for details).

**Figure 6:**
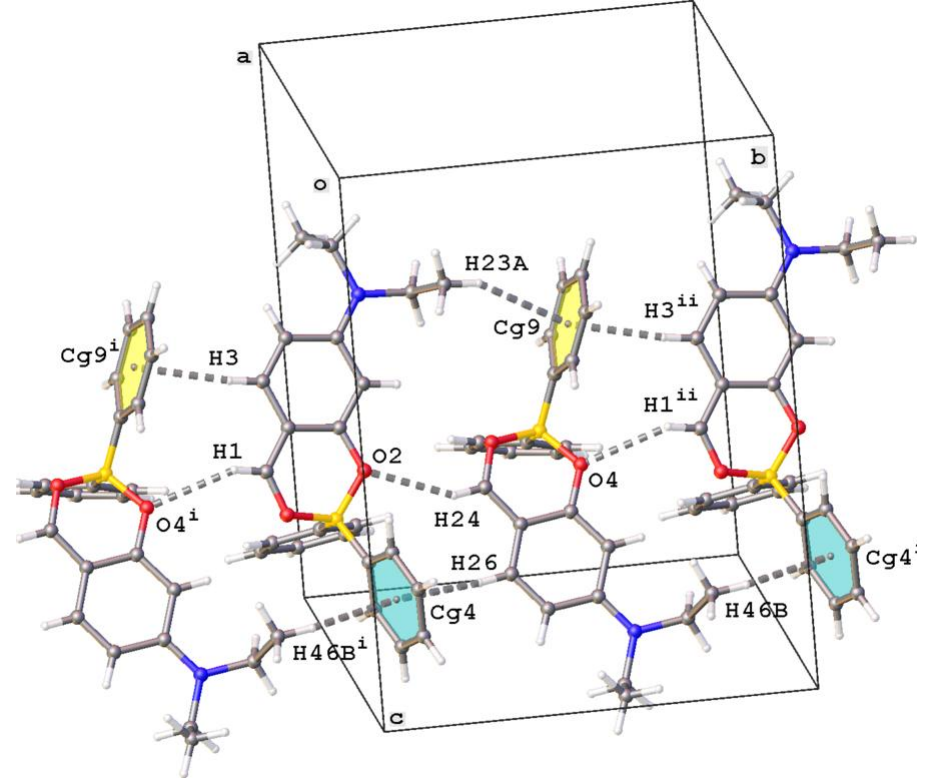
Chain of mol­ecules running in the *b*-axis direction in the crystal packing of (**I**). The C—H⋯O and C—H⋯π hydrogen bonds are shown as gray dashed lines. Symmetry codes: (i) *x*, −1 + *y*, *z*; (ii) *x*, 1 + *y*, *z. *Cg** 4 and *Cg*9 are the centroids of rings C14–C19 and C37–C42, respectively.

For compound (**I**), both mol­ecules *A* and *B* are linked by a C24—H24⋯O2 hydrogen bond (Table 2 ▸). In addition, mol­ecules *A* and *B* inter­act further through C1—H1⋯O4^i^ hydrogen bonds (see Table 2 ▸ for details). This builds a chain of alternating *A* and *B* mol­ecules running in the *b*-axis direction (Fig. 6 ▸). Within this chain and between neighboring chains several C—H⋯π inter­actions occur (Table 2 ▸), but π–π inter­actions are not present.

## 4. Database survey

**Figure 7:**
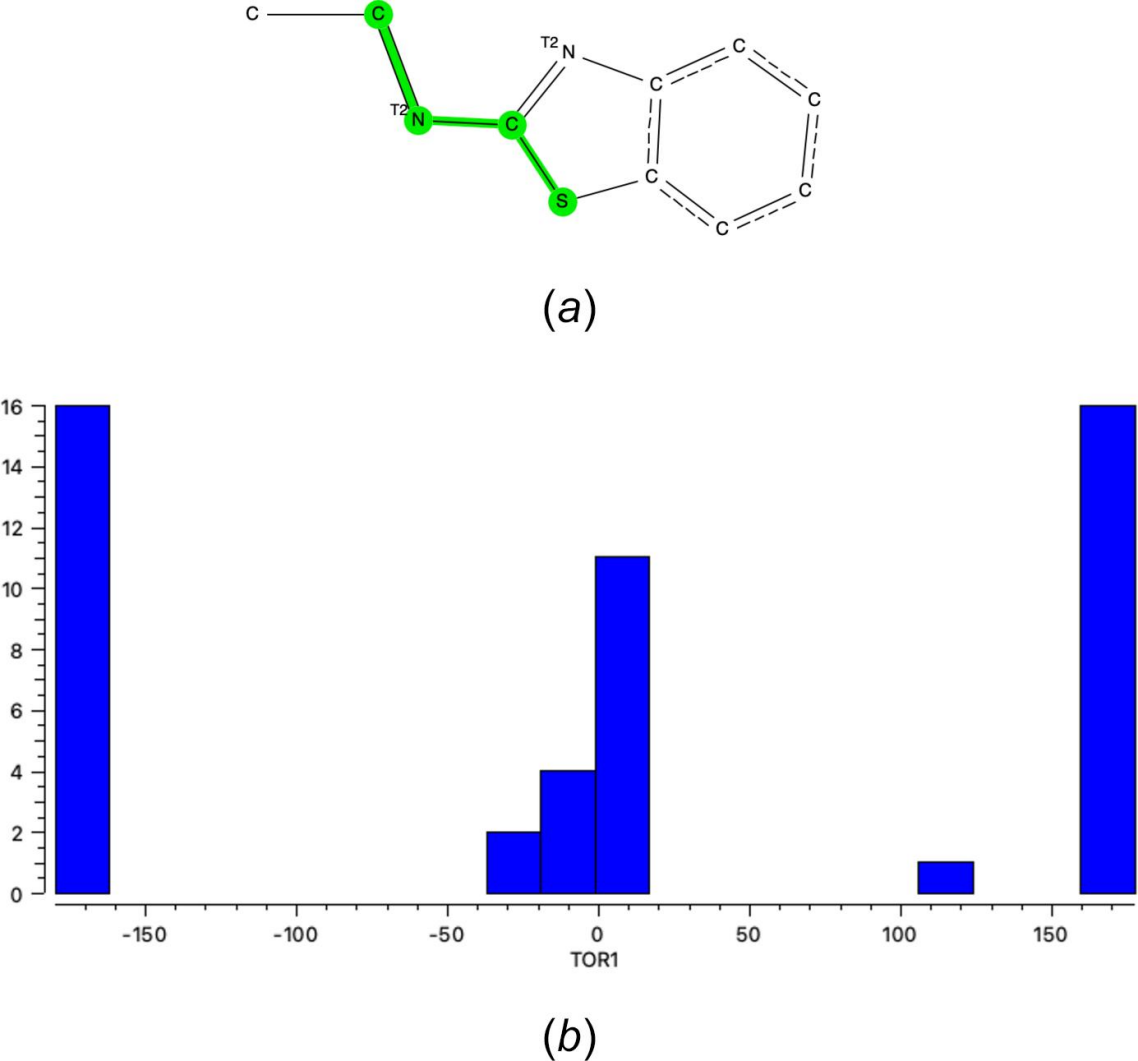
(*a*) Search fragment used in Conquest to perform the CSD survey. (*b*) The distribution of the torsion angle S—C—N—C in the search fragment shown as a histogram.

A search of the Cambridge Structural Database (CSD, Version 5.44, update of September 2023; Groom *et al.*, 2016 ▸) for the benzo­thia­zole fragment shown in Fig. 7 ▸ *a* gave 39 hits. For the majority of the hits (32 out of 50 values) the S—C—N=C torsion angle averages around ±180° (±*ap* or *trans*), while for 17 entries this torsion angle is close to 0° (±*sp* or *cis*; see Fig. 7 ▸ *b*). For one entry (refcode UXIRIE; Sović *et al.*, 2016 ▸), the unusual value of 121.0° (+*ac*) is caused by the incorporation of the terminal C—C bond of the search fragment into an indole ring. For **Et_2_N-Bz** this torsion angle is 177.55 (15)° for the major component of the benzo­thia­zole ring and −2.6 (4)° for the minor component.

A search for crystal structures containing a 1,3-dioxa-2-borata-1,2,3,4-tetra­hydro­naphthalene fragment in which the boron atom bears two additional carbon atoms resulted in five hits with refcodes ALUBOA (Light *et al.*, 2016 ▸), PEWLOS (Kliegel *et al.*, 1993 ▸), PUSBIO (Kliegel *et al.*, 1997 ▸), SEZGEJ and SEZGIN (Kliegel *et al.*, 1989 ▸). For all hits, the two carbon substituents are two phenyl groups. In these crystal structures, the 1,3-dioxa-2-borata-1,2,3,4-tetra­hydro­naphthalene also exhibits an envelope conformation with the boron atom as the flap and deviating from the best plane between 0.139 Å (PUSBIO) and 0.556 Å (PEWLOS). The average B—C(phen­yl) distance is 1.615 (7) Å [1.602 (4) Å in (**I**)]. The average B—O(phen­yl) and B—O(alk­yl) distances are 1.498 (10) and 1.543 (30) Å, respectively [1.507 (3) Å and 1.560 (3) Å in (**I**)].

## 5. Photophysical properties

**Figure 8:**
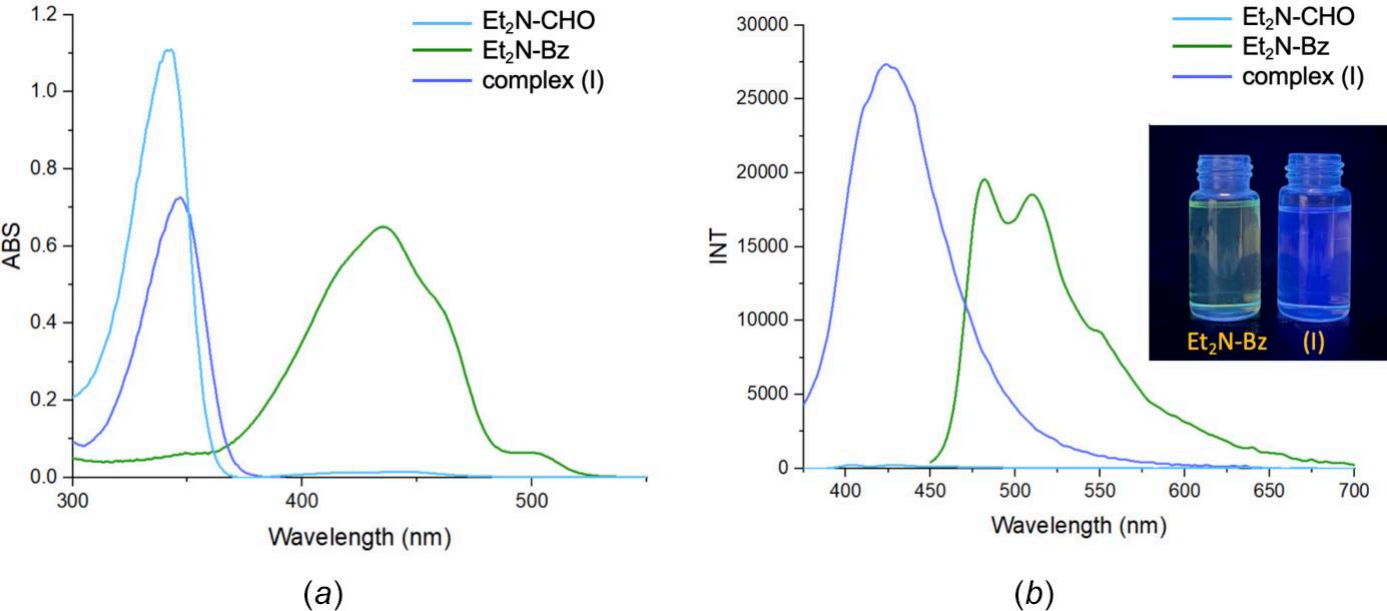
(*a*) UV–Vis absorption and (*b*) emission spectra of the examined compounds (10 µ*M* in CHCl_3_, λ_ex_ = 365 nm).

The UV–vis absorption and emission properties of the compounds **Et_2_N-CHO, Et_2_N**-**Bz**, and complex (**I**) at 10 µ*M* in chloro­form solvent are shown in Fig. 8 ▸ and Table 3 ▸. Accordingly, it can be seen that **Et_2_N-Bz** absorbs at 436 nm, while complex (**I**) shows absorption at 347 nm, which is a small shift from that of **Et_2_N-CHO** (343 nm). The absorption peaks (343 nm and 347 nm) are attributed to the π–π* transition of the aromatic ring. Under a UV lamp with a 365 nm excitation wavelength, a solution of **Et_2_N-Bz** fluoresces green, while a solution of complex (**I**) shifts towards blue. In addition, this complex exhibits a longer emission wavelength and greater fluorescence intensity than that of **Et_2_N-CHO**, demonstrating that complexation with boron can improve fluorescence properties compared to the free ligand.

**Figure 9:**
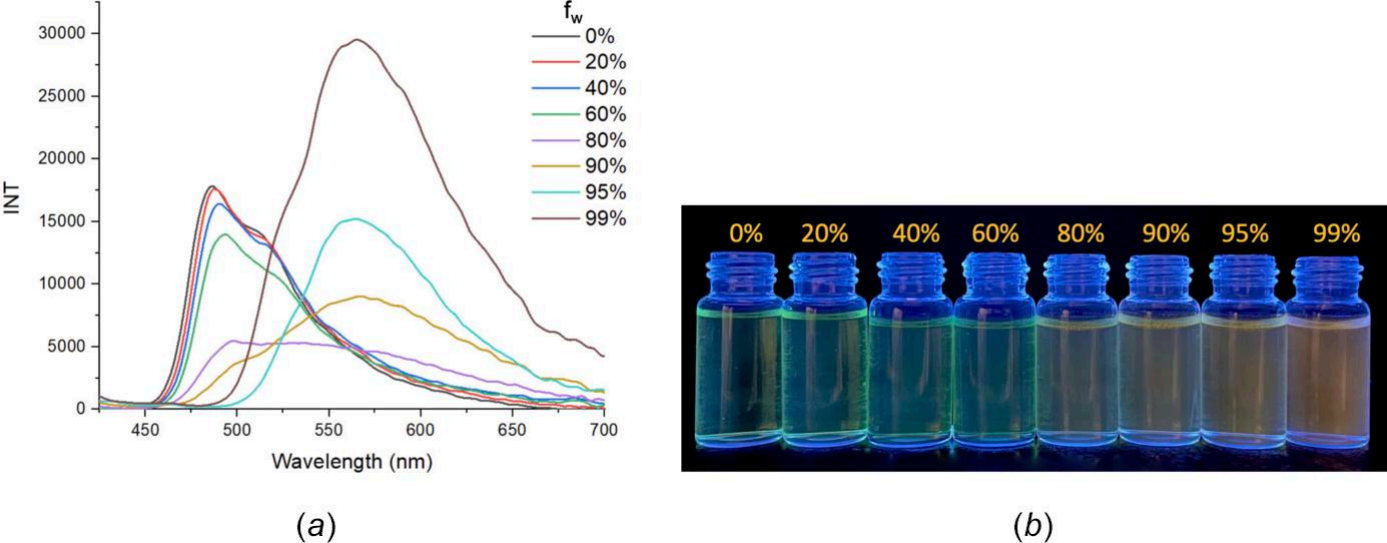
(*a*) Photoluminescence spectra and (*b*) fluorescent color change of compound **Et_2_N-Bz** at 10 µ*M* in different fractions of water in a MeOH–water mixture.

To investigate the AIE (aggregation-induced emission) properties of **Et_2_N-Bz** and (**I**), we recorded the emission spectra of their 10 µ*M* solutions in different fractions of water in a MeOH–water mixture. The results show that only compound **Et_2_N-Bz** is present as AIE active material (Fig. 9 ▸ and Fig. S1). The fluorescence color change from 0% to 99% water in the MeOH–water mixture from green to yellow is easily observed under a 365 nm UV lamp. The λ_em_ of **Et_2_N-Bz** in AIE spectra increases as the water fraction increases. This phenomenon can be explained by the fact that the solubility of **Et_2_N-Bz** decreased when the water ratio increased, which shortened the distance between mol­ecules and π–π stacking inter­actions appeared (Fig. 4 ▸), which affected the electron density in the mol­ecule, thus the emission wavelength and the emission intensity also changed (Hong *et al.*, 2009 ▸).

## 6. Synthesis and crystallization

**Synthesis of (** ***E*** **)-2-[(benzo[** ***d*** **]thia­zol-2-yl­imino)­meth­yl]-5-(di­ethyl­amino)­phenol (Et_2_N-Bz).**

A solution of 4-(di­ethyl­amino)-2-hy­droxy­benzaldehyde (193 mg, 1.0 mmol) and benzo[*d*]thia­zol-2-amine (150 mg, 1.0 mmol) in 10 mL of ethanol in a pressure tube was stirred at 348 K for 5 h. After cooling to room temperature (RT), the brown–yellow precipitated powder was filtered off, washed consecutively with a cold ethanol (1 × 5 mL), diethyl ether (1 × 5 mL) and then dried under vacuum at 323 K for 3 h. The yield was 87% (283 mg, 0.87 mmol). Single crystals suitable for X-ray diffraction and other analysis were obtained by slow evaporation within 8 h from a concentrated chloro­form/ethanol (2:1 *v*/*v*) solution at RT. ^1^H NMR (CDCl_3_, 600 MHz): δ 12.71 (*s*, 1H, OH), 8.96 (*s*, 1H, H_imine_), 7.88 (*dd*, ^3^ *J* = 8.4 Hz, ^4^ *J* = 0.6 Hz, 1H, Ar-H), 7.77 (*dd*, ^3^ *J* = 8.4 Hz, ^4^ *J* = 0.6 Hz, 1H, Ar-H), 7.43 (*m* 1H, Ar-H), 7.30 (*m* 1H, Ar-H), 7.26 (*d*, ^3^ *J* = 8.4 Hz, 1H, Ar-H), 6.31 (*dd*, ^3^ *J* = 8.4 Hz, ^4^ *J* = 2.4 Hz, 1H, Ar-H), 6.19 (*d*, ^4^ *J* = 2.4 Hz, 1H, Ar-H), 3.43 (*q*, ^3^ *J* = 7.2 Hz, 4H, C*H_2_* CH_3_), 1.23 (*t*, ^3^ *J* = 7.2 Hz, 6H, CH_2_C*H_3_*).

**Reaction of (** ***E*** **)-2-[(benzo[** ***d*** **]thia­zol-2-yl­imino)­meth­yl]-5-(di­ethyl­amino)­phenol (Et_2_N-Bz) with tri­phenyl­borane**.

***In chloro­form***: A solution of compound **Et_2_N-Bz** (65 mg, 0.2 mmol) and BPh_3_ (73 mg, 0.30 mmol) in 3 mL of chloro­form in a pressure tube was stirred at 333 K for 24 h. After cooling down, single crystals suitable for X-ray diffraction and other analysis were obtained by slow evaporation within 8 h from a reaction solution at RT. The yield was 55% (39.30 mg, 0.11 mmol). ^1^H NMR (CDCl_3_, 600 MHz): δ 9,49 (*s*, 1H, CHO), 7,81 (*d*, ^3^ *J* = 5.5 Hz, 1H, Ar-H), 7.53 (*m*, 3H, Ar-H), 7.46 (*m*, 4H, Ar-H), 7.26 (*m*, 2H, Ar-H _l_), 6.27 (*dd*, ^3^ *J* = 5.5 Hz, ^4^ *J* = 2.0 Hz, 1H, Ar-H), 6.07 (*d*, ^4^ *J* = 2.0 Hz, 1H, Ar-H), 3.41 (*q*, ^3^ *J* = 6.0 Hz, 4H, C*H_2_* CH_3_), 1.21 (*t*, ^3^ *J* = 6.0 Hz, 6H, CH_2_C*H_3_*).

***In other solvents*** **:** The experiments in other solvents such as toluene, THF, ethanol were conducted under the same conditions as in chloro­form. The course of reaction was monitored by TLC analysis. The results indicated that no new products were formed after 24 h of reaction.

**Reaction of 4-(di­ethyl­amino)-2-hy­droxy­benzaldehyde (Et_2_N-CHO) with tri­phenyl­borane.** A solution of 4-(di­ethyl­amino)-2-hy­droxy­benzaldehyde (97 mg, 0.5 mmol) and BPh_3_ (182 mg, 0.75 mmol) in 3 mL of chloro­form in a pressure tube was stirred at 333 K for 24 h. The TLC analysis results indicated that there was no signal of the new product.

## 7. Refinement

Crystal data, data collection and structure refinement details are summarized in Table 4 ▸. All H atoms bonded to C atoms were placed in idealized positions and refined using a riding model with C—H distances of 0.93 (aromatic), 0.97 (CH_2_) and 0.96 Å (CH_3_). Non-hydrogen atoms were refined anisotropically and hydrogen atoms with isotropic temperature factors fixed at 1.2 times *U*_eq_ of the parent atoms (1.5 for methyl groups). For the O—H group in **Et_2_N-Bz**, the *SHELXL* command AFIX 148 was used in combination with *U*(H) = 1.2*U*_eq_(O). One of the ethyl groups in **Et_2_N-Bz** is disordered over two sets of sites with refined occupancies of 0.822 (5) and 0.178 (5). Also the benzo­thia­zole group is disordered over two positions by a rotation of 180° resulting in refined occupancies of 0.843 (2) and 0.157 (2) for atoms S1 and N3.

## Supplementary Material

Crystal structure: contains datablock(s) Et2N-Bz, I. DOI: 10.1107/S2056989023008514/pk2698sup1.cif

Structure factors: contains datablock(s) Et2N-Bz. DOI: 10.1107/S2056989023008514/pk2698Et2N-Bzsup2.hkl

Structure factors: contains datablock(s) I. DOI: 10.1107/S2056989023008514/pk2698Isup3.hkl

Supporting information file. DOI: 10.1107/S2056989023008514/pk2698Et2N-Bzsup4.cml

Fig. S1. DOI: 10.1107/S2056989023008514/pk2698sup5.png

CCDC references: 2297768, 2297767

Additional supporting information:  crystallographic information; 3D view; checkCIF report

## Figures and Tables

**Table 1:** Hydrogen-bond geometry (Å, °) for **Et_2_N-Bz** [Chem scheme1]

*D*—H⋯*A*	*D*—H	H⋯*A*	*D*⋯*A*	*D*—H⋯*A*
O18—H18⋯N10	0.90 (3)	1.79 (3)	2.582 (2)	147 (1)
C16—H16⋯O18^i^	0.93	2.48	3.312 (3)	149

Symmetry code: (i) 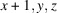 .

**Table 2:** Hydrogen-bond geometry (Å, °) for (**I**) *Cg*3, *Cg*4, *Cg*7, *Cg*8 and *Cg*9 are the centroids of rings C8–C13, C14–C19, C25–C30, C31–C36 and C37–C42, respectively.

*D*—H⋯*A*	*D*—H	H⋯*A*	*D*⋯*A*	*D*—H⋯*A*
C24—H24⋯O2	0.93	2.51	3.343 (3)	149
C1—H1⋯O4^i^	0.93	2.55	3.334 (3)	142
C3—H3⋯*Cg*9^i^	0.93	2.59	3.510 (4)	169
C23—H23*A*⋯*Cg*9	0.96	2.88	3.771 (5)	155
C26—H26⋯*Cg*4	0.93	2.66	3.562 (3)	164
C46—H46*B*⋯*Cg*4^ii^	0.96	2.69	3.623 (4)	164
C21—H21*A*⋯*Cg*3^iii^	0.96	2.87	3.815 (5)	168
C43—H43*B*⋯*Cg*7^iv^	0.96	2.93	3.626 (3)	129
C44—H44*C*⋯*Cg*8^iv^	0.96	2.95	3.876 (4)	162

Symmetry codes: (i) 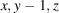 ; (ii) 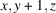 ; (iii) 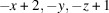 ; (iv) 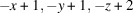 .

**Table 3:** Photophysical data for the examined compounds (in CHCl_3_, 10 µ*M*)

Compound	Absorption	Emission		Stokes shift
	λ_ABS_ (nm) / (ɛ 10^3^ *M* ^−1^.cm^−1^)	λ_em_ (nm)	Intensity (a.u.)	Δν (cm^−1^)
**Et_2_N-CHO**	343 (111)	425	224	–
**Et_2_N-Bz**	436 (65); 504 (5)	481 / 510	19533 / 18516	3327
complex (**I**)	347 (73)	432	27349	5670

**Table 4:** Experimental details

	(**I**)	**Et_2_N-Bz**
Crystal data		
Chemical formula	C_23_H_24_BNO_2_	C_18_H_19_N_3_OS
*M*_r_	362.24	325.42
Crystal system, space group	Triclinic, *P* 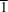	Monoclinic, *P*2_1_/*n*
Temperature (K)	293	294
*a*, *b*, *c* (Å)	10.6725 (5), 11.8934 (4), 16.1411 (7)	7.2881 (2), 22.0453 (8), 10.2761 (3)
α, β, γ (°)	86.207 (3), 87.553 (4), 88.394 (3)	90, 90.280 (3), 90
*V* (Å^3^)	2041.82 (15)	1651.02 (9)
*Z*	4	4
Radiation type	Mo *K*α	Mo *K*α
μ (mm^−1^)	0.07	0.20
Crystal size (mm)	0.5 × 0.15 × 0.15	0.45 × 0.3 × 0.05

Data collection		
Diffractometer	SuperNova, Single source at offset/far, Eos	SuperNova, Single source at offset/far, Eos
Absorption correction	Multi-scan (*CrysAlis PRO*; Rigaku OD, 2022 ▸)	Multi-scan (*CrysAlis PRO*; Rigaku OD, 2022 ▸)
*T*_min_, *T*_max_	0.839, 1.000	0.395, 1.000
No. of measured, independent and observed [*I* > 2σ(*I*)] reflections	33181, 8340, 4623	33381, 3366, 2650
*R*_int_	0.046	0.039
(sin θ/λ)_max_ (Å^−1^)	0.625	0.625

Refinement		
*R*[*F* ^2^ > 2σ(*F* ^2^)], *wR*(*F* ^2^), *S*	0.069, 0.196, 1.05	0.053, 0.138, 1.03
No. of reflections	8340	3366
No. of parameters	491	227
No. of restraints	0	5
H-atom treatment	H-atom parameters constrained	H-atom parameters constrained
Δρ_max_, Δρ_min_ (e Å^−3^)	0.18, −0.18	0.40, −0.27

Computer programs: *CrysAlis PRO* (Rigaku OD, 2022 ▸), *SHELXT2014/5* (Sheldrick, 2015*a* ▸), *SHELXL2016/4* (Sheldrick, 2015*b* ▸) and *OLEX2* 1.3 (Dolomanov *et al.*, 2009 ▸).

## supplementary crystallographic information

### (E)-2-[(Benzo[d]thiazol-2-ylimino)methyl]-5-(diethylamino)phenol (Et2N-Bz) Crystal data

**Table:**

C_18_H_19_N_3_OS	*F*(000) = 688
*M**_r_* = 325.42	*D*_x_ = 1.309 Mg m^−^^3^
Monoclinic, *P*2_1_/*n*	Mo *K*α radiation, λ = 0.71073 Å
*a* = 7.2881 (2) Å	Cell parameters from 10518 reflections
*b* = 22.0453 (8) Å	θ = 2.9–28.1°
*c* = 10.2761 (3) Å	µ = 0.20 mm^−^^1^
β = 90.280 (3)°	*T* = 294 K
*V* = 1651.02 (9) Å^3^	Plate, orange
*Z* = 4	0.45 × 0.3 × 0.05 mm

### (E)-2-[(Benzo[d]thiazol-2-ylimino)methyl]-5-(diethylamino)phenol (Et2N-Bz) Data collection

**Table:**

SuperNova, Single source at offset/far, Eos diffractometer	3366 independent reflections
Radiation source: micro-focus sealed X-ray tube, SuperNova (Mo) X-ray Source	2650 reflections with *I* > 2σ(*I*)
Mirror monochromator	*R*_int_ = 0.039
Detector resolution: 15.9631 pixels mm^-1^	θ_max_ = 26.4°, θ_min_ = 2.7°
ω scans	*h* = −9→9
Absorption correction: multi-scan (CrysAlisPro; Rigaku OD, 2022)	*k* = −27→27
*T*_min_ = 0.395, *T*_max_ = 1.000	*l* = −12→12
33381 measured reflections	

### (E)-2-[(Benzo[d]thiazol-2-ylimino)methyl]-5-(diethylamino)phenol (Et2N-Bz) Refinement

**Table:**

Refinement on *F*^2^	Primary atom site location: dual
Least-squares matrix: full	Hydrogen site location: inferred from neighbouring sites
*R*[*F*^2^ > 2σ(*F*^2^)] = 0.053	H-atom parameters constrained
*wR*(*F*^2^) = 0.138	*w* = 1/[σ^2^(*F*_o_^2^) + (0.0472*P*)^2^ + 1.010*P*] where *P* = (*F*_o_^2^ + 2*F*_c_^2^)/3
*S* = 1.03	(Δ/σ)_max_ < 0.001
3366 reflections	Δρ_max_ = 0.40 e Å^−^^3^
227 parameters	Δρ_min_ = −0.27 e Å^−^^3^
5 restraints	

### (E)-2-[(Benzo[d]thiazol-2-ylimino)methyl]-5-(diethylamino)phenol (Et2N-Bz) Special details

**Table:**

Geometry. All esds (except the esd in the dihedral angle between two l.s. planes) are estimated using the full covariance matrix. The cell esds are taken into account individually in the estimation of esds in distances, angles and torsion angles; correlations between esds in cell parameters are only used when they are defined by crystal symmetry. An approximate (isotropic) treatment of cell esds is used for estimating esds involving l.s. planes.

### (E)-2-[(Benzo[d]thiazol-2-ylimino)methyl]-5-(diethylamino)phenol (Et2N-Bz) Fractional atomic coordinates and isotropic or equivalent isotropic displacement parameters (Å^2^)

**Table:**

	*x*	*y*	*z*	*U*_iso_*/*U*_eq_	Occ. (<1)
S1	0.22145 (11)	0.35862 (4)	0.49674 (8)	0.0589 (3)	0.843 (2)
N3	0.5480 (4)	0.33979 (12)	0.4072 (3)	0.0509 (6)	0.843 (2)
S1B	0.6141 (8)	0.3376 (3)	0.3866 (6)	0.0589 (3)	0.157 (2)
N3B	0.2914 (14)	0.3507 (6)	0.4767 (13)	0.0509 (6)	0.157 (2)
C2	0.4561 (3)	0.37408 (9)	0.48618 (19)	0.0476 (5)	
C4	0.4325 (3)	0.29811 (10)	0.3439 (2)	0.0555 (6)	
C5	0.4808 (4)	0.25480 (12)	0.2522 (3)	0.0741 (8)	
H5	0.601860	0.251192	0.225175	0.089*	
C6	0.3495 (6)	0.21786 (14)	0.2026 (3)	0.0910 (10)	
H6	0.381988	0.188890	0.141123	0.109*	
C7	0.1721 (6)	0.22200 (14)	0.2403 (3)	0.0915 (10)	
H7	0.086004	0.195763	0.204073	0.110*	
C8	0.1154 (4)	0.26439 (14)	0.3316 (3)	0.0819 (8)	
H8	−0.006568	0.267126	0.357211	0.098*	
C9	0.2508 (4)	0.30300 (11)	0.3837 (2)	0.0599 (6)	
N10	0.5154 (2)	0.42027 (8)	0.56646 (16)	0.0485 (4)	
C11	0.6886 (3)	0.43504 (9)	0.57170 (19)	0.0466 (5)	
H11	0.771560	0.414636	0.518984	0.056*	
C12	0.7534 (3)	0.48131 (9)	0.65551 (18)	0.0426 (5)	
C13	0.6334 (3)	0.51562 (9)	0.7349 (2)	0.0441 (5)	
C14	0.6985 (3)	0.55958 (10)	0.8176 (2)	0.0487 (5)	
H14	0.615934	0.581285	0.868238	0.058*	
C15	0.8867 (3)	0.57262 (10)	0.8276 (2)	0.0507 (5)	
C16	1.0081 (3)	0.53789 (11)	0.7490 (2)	0.0566 (6)	
H16	1.133804	0.544747	0.754032	0.068*	
C17	0.9420 (3)	0.49499 (11)	0.6671 (2)	0.0519 (5)	
H17	1.024681	0.473532	0.616078	0.062*	
O18	0.45128 (19)	0.50579 (8)	0.73184 (17)	0.0593 (4)	
H18	0.4263 (9)	0.4748 (14)	0.678 (3)	0.089*	
N19	0.9503 (3)	0.61697 (10)	0.9084 (2)	0.0656 (6)	
C20	1.1470 (4)	0.63271 (18)	0.9180 (4)	0.0757 (11)	0.822 (5)
H20A	1.159741	0.674393	0.946869	0.091*	0.822 (5)
H20B	1.203061	0.629211	0.832933	0.091*	0.822 (5)
C20B	1.1347 (19)	0.6014 (8)	0.9781 (17)	0.0757 (11)	0.178 (5)
H20C	1.127550	0.606973	1.071569	0.091*	0.178 (5)
H20D	1.175630	0.560508	0.958830	0.091*	0.178 (5)
C21	1.2439 (5)	0.5911 (2)	1.0127 (4)	0.1063 (16)	0.822 (5)
H21A	1.236429	0.550100	0.981755	0.159*	0.822 (5)
H21B	1.186608	0.593961	1.096348	0.159*	0.822 (5)
H21C	1.370379	0.602874	1.020040	0.159*	0.822 (5)
C21B	1.253 (2)	0.6487 (10)	0.9142 (18)	0.1063 (16)	0.178 (5)
H21D	1.260491	0.640653	0.822578	0.159*	0.178 (5)
H21E	1.374032	0.647580	0.951688	0.159*	0.178 (5)
H21F	1.200460	0.688137	0.927649	0.159*	0.178 (5)
C22	0.8250 (3)	0.65065 (12)	0.9929 (2)	0.0645 (7)	
H22A	0.714448	0.660161	0.944081	0.077*	
H22B	0.882488	0.688693	1.017338	0.077*	
C23	0.7730 (4)	0.61722 (15)	1.1136 (3)	0.0833 (9)	
H23A	0.714527	0.579706	1.090375	0.125*	
H23B	0.689985	0.641523	1.163662	0.125*	
H23C	0.881184	0.608942	1.164225	0.125*	

### (E)-2-[(Benzo[d]thiazol-2-ylimino)methyl]-5-(diethylamino)phenol (Et2N-Bz) Atomic displacement parameters (Å^2^)

**Table:**

	*U*^11^	*U*^22^	*U*^33^	*U*^12^	*U*^13^	*U*^23^
S1	0.0620 (5)	0.0571 (5)	0.0577 (5)	−0.0060 (4)	0.0067 (4)	−0.0146 (3)
N3	0.0554 (16)	0.0500 (14)	0.0473 (14)	0.0042 (13)	−0.0004 (12)	0.0006 (10)
S1B	0.0620 (5)	0.0571 (5)	0.0577 (5)	−0.0060 (4)	0.0067 (4)	−0.0146 (3)
N3B	0.0554 (16)	0.0500 (14)	0.0473 (14)	0.0042 (13)	−0.0004 (12)	0.0006 (10)
C2	0.0601 (13)	0.0421 (11)	0.0404 (10)	0.0015 (9)	−0.0025 (9)	0.0023 (9)
C4	0.0757 (16)	0.0406 (12)	0.0501 (12)	0.0028 (11)	−0.0060 (11)	0.0037 (10)
C5	0.100 (2)	0.0544 (15)	0.0685 (16)	0.0111 (14)	0.0075 (15)	−0.0057 (13)
C6	0.144 (3)	0.0584 (17)	0.0705 (18)	−0.0063 (19)	0.009 (2)	−0.0179 (14)
C7	0.128 (3)	0.073 (2)	0.0736 (19)	−0.035 (2)	−0.0106 (19)	−0.0159 (16)
C8	0.0814 (19)	0.088 (2)	0.0765 (18)	−0.0213 (16)	0.0033 (15)	0.0036 (16)
C9	0.0785 (17)	0.0535 (14)	0.0478 (12)	−0.0060 (12)	−0.0003 (11)	0.0006 (10)
N10	0.0563 (11)	0.0436 (10)	0.0455 (9)	−0.0018 (8)	−0.0026 (8)	−0.0019 (8)
C11	0.0555 (12)	0.0435 (11)	0.0409 (10)	0.0086 (9)	0.0009 (9)	0.0028 (9)
C12	0.0442 (11)	0.0435 (11)	0.0402 (10)	0.0050 (8)	−0.0002 (8)	0.0013 (8)
C13	0.0372 (10)	0.0465 (11)	0.0484 (11)	0.0013 (8)	0.0006 (8)	0.0006 (9)
C14	0.0408 (11)	0.0538 (13)	0.0516 (12)	0.0051 (9)	0.0031 (9)	−0.0079 (10)
C15	0.0432 (11)	0.0589 (13)	0.0499 (12)	0.0006 (10)	−0.0027 (9)	−0.0063 (10)
C16	0.0357 (10)	0.0760 (16)	0.0579 (13)	0.0012 (10)	0.0019 (9)	−0.0095 (12)
C17	0.0423 (11)	0.0642 (14)	0.0492 (11)	0.0090 (10)	0.0065 (9)	−0.0046 (10)
O18	0.0365 (8)	0.0677 (11)	0.0737 (11)	−0.0037 (7)	0.0048 (7)	−0.0206 (9)
N19	0.0486 (11)	0.0791 (15)	0.0689 (13)	−0.0058 (10)	−0.0028 (9)	−0.0229 (11)
C20	0.0567 (18)	0.090 (3)	0.081 (3)	−0.0225 (19)	0.0038 (18)	−0.022 (2)
C20B	0.0567 (18)	0.090 (3)	0.081 (3)	−0.0225 (19)	0.0038 (18)	−0.022 (2)
C21	0.059 (2)	0.181 (5)	0.079 (2)	0.019 (3)	−0.0123 (19)	−0.016 (3)
C21B	0.059 (2)	0.181 (5)	0.079 (2)	0.019 (3)	−0.0123 (19)	−0.016 (3)
C22	0.0664 (15)	0.0600 (15)	0.0670 (15)	−0.0030 (12)	−0.0060 (12)	−0.0161 (12)
C23	0.085 (2)	0.099 (2)	0.0665 (17)	0.0015 (17)	−0.0003 (14)	−0.0042 (16)

### (E)-2-[(Benzo[d]thiazol-2-ylimino)methyl]-5-(diethylamino)phenol (Et2N-Bz) Geometric parameters (Å, º)

**Table:**

S1c—C2	1.748 (2)	C7—C8	1.389 (4)
S1Bd—C2	1.741 (5)	C8—H8	0.9300
N3c—C2	1.298 (3)	C8—C9	1.407 (4)
N3Bd—C2	1.310 (9)	N10—C11	1.304 (3)
S1Bd—C4	1.642 (6)	C11—H11	0.9300
N3c—C4	1.403 (3)	C11—C12	1.415 (3)
S1c—C9	1.703 (2)	C12—C13	1.418 (3)
N3Bd—C9	1.450 (9)	C12—C17	1.412 (3)
C20a—H20A	0.9700	C13—C14	1.372 (3)
C20a—H20B	0.9700	C13—O18	1.345 (2)
C20Bb—H20C	0.9700	C14—H14	0.9300
C20Bb—H20D	0.9700	C14—C15	1.405 (3)
C20a—C21	1.510 (6)	C15—C16	1.424 (3)
C21a—H21A	0.9600	C15—N19	1.363 (3)
C21a—H21B	0.9600	C16—H16	0.9300
C21a—H21C	0.9600	C16—C17	1.353 (3)
C20Bb—C21B	1.508 (17)	C17—H17	0.9300
C21Bb—H21D	0.9600	O18—H18	0.90 (3)
C21Bb—H21E	0.9600	N19—C20	1.477 (4)
C21Bb—H21F	0.9600	N19—C20B	1.558 (15)
C2—N10	1.379 (3)	N19—C22	1.465 (3)
C4—C5	1.388 (3)	C22—H22A	0.9700
C4—C9	1.392 (3)	C22—H22B	0.9700
C5—H5	0.9300	C22—C23	1.493 (4)
C5—C6	1.354 (4)	C23—H23A	0.9600
C6—H6	0.9300	C23—H23B	0.9600
C6—C7	1.355 (5)	C23—H23C	0.9600
C7—H7	0.9300		
			
C21Bb—C20Bb—N19	98.2 (13)	C8—C9—S1	127.2 (2)
N10—C2—S1	114.26 (15)	C8—C9—N3B	146.0 (5)
N10—C2—S1B	119.1 (3)	C9—S1c—C2	88.38 (12)
C21a—C20a—H20A	109.5	C11—N10—C2	120.64 (19)
H20Aa—C20a—H20B	108.1	N10—C11—H11	119.1
C21a—C20a—H20B	109.5	N10—C11—C12	121.70 (19)
C21Bb—C20Bb—H20C	112.1	C12—C11—H11	119.1
C21Bb—C20Bb—H20D	112.1	C11—C12—C13	121.98 (19)
H20Cb—C20Bb—H20D	109.8	C17—C12—C11	121.86 (19)
C20a—C21a—H21A	109.5	C17—C12—C13	116.15 (18)
C20a—C21a—H21B	109.5	C14—C13—C12	121.39 (18)
H21Aa—C21a—H21B	109.5	O18—C13—C12	120.82 (18)
H21Ba—C21a—H21C	109.5	O18—C13—C14	117.79 (18)
C20a—C21a—H21C	109.5	C13—C14—H14	119.2
H21Aa—C21a—H21C	109.5	C13—C14—C15	121.63 (19)
C20Bb—C21Bb—H21D	109.5	C15—C14—H14	119.2
H21Db—C21Bb—H21E	109.5	C14—C15—C16	117.2 (2)
C20Bb—C21Bb—H21E	109.5	N19—C15—C14	121.4 (2)
C20Bb—C21Bb—H21F	109.5	N19—C15—C16	121.38 (19)
H21Db—C21Bb—H21F	109.5	C15—C16—H16	119.7
H21Eb—C21Bb—H21F	109.5	C17—C16—C15	120.6 (2)
N3c—C2—S1	115.66 (19)	C17—C16—H16	119.7
N3Bd—C2—S1B	112.5 (5)	C12—C17—H17	118.5
N3c—C2—N10	130.1 (2)	C16—C17—C12	123.0 (2)
N3Bd—C2—N10	128.3 (5)	C16—C17—H17	118.5
C2—N3c—C4	111.2 (3)	C13—O18—H18	109.5
C2—N3Bd—C9	121.2 (8)	C15—N19—C20	122.4 (2)
C4—S1Bd—C2	82.4 (3)	C15—N19—C20B	114.3 (7)
C5—C4—N3	127.7 (3)	C15—N19—C22	120.89 (19)
C5—C4—S1B	109.8 (3)	C22—N19—C20	116.7 (2)
C5—C4—C9	119.8 (2)	N19—C20a—H20A	109.5
C9—C4—N3	112.5 (2)	N19—C20a—H20B	109.5
C9—C4—S1B	130.3 (3)	N19—C20Bb—H20C	112.1
C4—C5—H5	120.4	N19—C20Bb—H20D	112.1
C6—C5—C4	119.2 (3)	N19—C20a—C21	110.6 (3)
C6—C5—H5	120.4	C22—N19—C20B	112.2 (7)
C5—C6—H6	119.2	N19—C22—H22A	108.8
C5—C6—C7	121.7 (3)	N19—C22—H22B	108.8
C7—C6—H6	119.2	N19—C22—C23	113.8 (2)
C6—C7—H7	119.1	H22A—C22—H22B	107.7
C6—C7—C8	121.8 (3)	C23—C22—H22A	108.8
C8—C7—H7	119.1	C23—C22—H22B	108.8
C7—C8—H8	121.5	C22—C23—H23A	109.5
C7—C8—C9	117.0 (3)	C22—C23—H23B	109.5
C9—C8—H8	121.5	C22—C23—H23C	109.5
C4—C9—S1	112.27 (18)	H23A—C23—H23B	109.5
C4—C9—N3B	93.4 (5)	H23A—C23—H23C	109.5
C4—C9—C8	120.5 (2)	H23B—C23—H23C	109.5
			
C4—N3c—C2—N10	179.9 (2)	S1Bd—C4—C9—C8	178.8 (4)
N3c—C4—C9—S1c	0.5 (3)	C2—N3Bd—C9—C8	−178.8 (4)
C5—C4—C9—S1c	179.79 (18)	C2—S1c—C9—C8	−179.8 (2)
C4—S1Bd—C2—N10	179.3 (2)	S1c—C2—N10—C11	177.55 (15)
C9—N3Bd—C2—N10	178.2 (6)	N3Bd—C2—N10—C11	174.4 (9)
C5—C4—C9—N3Bd	177.7 (6)	N3c—C2—N10—C11	−0.6 (4)
S1Bd—C4—C9—N3Bd	−3.4 (7)	S1Bd—C2—N10—C11	−2.6 (4)
C2—N10—C11—C12	−178.51 (18)	C20a—N19—C22—C23	−99.1 (3)
C4—S1Bd—C2—N3Bd	1.8 (9)	C20Bb—N19—C22—C23	−60.2 (7)
C4—N3c—C2—S1c	1.7 (3)	C9—C4—C5—C6	0.1 (4)
C9—S1c—C2—N10	−179.71 (16)	N10—C11—C12—C13	−2.3 (3)
C4—C5—C6—C7	−0.1 (5)	N10—C11—C12—C17	176.20 (19)
C5—C4—C9—C8	0.0 (4)	C11—C12—C13—C14	178.74 (19)
C5—C6—C7—C8	0.1 (5)	C11—C12—C13—O18	−0.9 (3)
C6—C7—C8—C9	−0.1 (5)	C11—C12—C17—C16	−178.2 (2)
C7—C8—C9—S1c	−179.8 (2)	C12—C13—C14—C15	0.0 (3)
C7—C8—C9—N3Bd	−175.9 (11)	C13—C12—C17—C16	0.4 (3)
C7—C8—C9—C4	0.0 (4)	C13—C14—C15—C16	−0.5 (3)
C14—C15—N19—C20a	−178.3 (3)	C13—C14—C15—N19	179.0 (2)
C9—S1c—C2—N3c	−1.3 (2)	C14—C15—C16—C17	1.1 (3)
C14—C15—N19—C20Bb	142.1 (7)	C14—C15—N19—C22	3.1 (4)
C16—C15—N19—C20a	1.3 (4)	C15—C16—C17—C12	−1.0 (4)
C16—C15—N19—C20Bb	−38.4 (8)	C15—N19—C20a—C21a	−84.6 (4)
C9—N3Bd—C2—S1Bd	−4.7 (15)	C15—N19—C20Bb—C21Bb	114.0 (11)
C2—N3c—C4—C5	179.3 (2)	C15—N19—C22—C23	79.6 (3)
C2—S1Bd—C4—C5	−179.7 (2)	C16—C15—N19—C22	−177.4 (2)
C2—S1Bd—C4—C9	1.3 (5)	C17—C12—C13—C14	0.1 (3)
C2—N3c—C4—C9	−1.4 (3)	C17—C12—C13—O18	−179.50 (19)
S1Bd—C4—C5—C6	−179.0 (3)	O18—C13—C14—C15	179.6 (2)
N3c—C4—C5—C6	179.3 (3)	N19—C15—C16—C17	−178.5 (2)
C2—S1c—C9—C4	0.37 (18)	C22—N19—C20Bb—C21Bb	−103.5 (12)
C2—N3Bd—C9—C4	4.7 (12)	C22—N19—C20a—C21a	94.1 (3)
N3c—C4—C9—C8	−179.3 (2)		

### (E)-2-[(Benzo[d]thiazol-2-ylimino)methyl]-5-(diethylamino)phenol (Et2N-Bz) Hydrogen-bond geometry (Å, º)

**Table:**

*D*—H···*A*	*D*—H	H···*A*	*D*···*A*	*D*—H···*A*
O18—H18···N10	0.90 (3)	1.79 (3)	2.582 (2)	147 (1)
C16—H16···O18^i^	0.93	2.48	3.312 (3)	149

Symmetry code: (i) *x*+1, *y*, *z*.

### 7-(Diethylamino)-2,2-diphenyl-1,3-dioxa-2-borata-1,2-dihydronaphthalene (I) Crystal data

**Table:**

C_23_H_24_BNO_2_	*Z* = 4
*M**_r_* = 362.24	*F*(000) = 760
Triclinic, *P*1	*D*_x_ = 1.162 Mg m^−^^3^
*a* = 10.6725 (5) Å	Mo *K*α radiation, λ = 0.71073 Å
*b* = 11.8934 (4) Å	Cell parameters from 7067 reflections
*c* = 16.1411 (7) Å	θ = 2.9–22.8°
α = 86.207 (3)°	µ = 0.07 mm^−^^1^
β = 87.553 (4)°	*T* = 293 K
γ = 88.394 (3)°	Needle, brown
*V* = 2041.82 (15) Å^3^	0.5 × 0.15 × 0.15 mm

### 7-(Diethylamino)-2,2-diphenyl-1,3-dioxa-2-borata-1,2-dihydronaphthalene (I) Data collection

**Table:**

SuperNova, Single source at offset/far, Eos diffractometer	8340 independent reflections
Radiation source: micro-focus sealed X-ray tube, SuperNova (Mo) X-ray Source	4623 reflections with *I* > 2σ(*I*)
Mirror monochromator	*R*_int_ = 0.046
Detector resolution: 15.9631 pixels mm^-1^	θ_max_ = 26.4°, θ_min_ = 2.5°
ω scans	*h* = −13→13
Absorption correction: multi-scan (CrysAlisPro; Rigaku OD, 2022)	*k* = −14→14
*T*_min_ = 0.839, *T*_max_ = 1.000	*l* = −20→20
33181 measured reflections	

### 7-(Diethylamino)-2,2-diphenyl-1,3-dioxa-2-borata-1,2-dihydronaphthalene (I) Refinement

**Table:**

Refinement on *F*^2^	Primary atom site location: dual
Least-squares matrix: full	Hydrogen site location: inferred from neighbouring sites
*R*[*F*^2^ > 2σ(*F*^2^)] = 0.069	H-atom parameters constrained
*wR*(*F*^2^) = 0.196	*w* = 1/[σ^2^(*F*_o_^2^) + (0.0636*P*)^2^ + 0.750*P*] where *P* = (*F*_o_^2^ + 2*F*_c_^2^)/3
*S* = 1.05	(Δ/σ)_max_ < 0.001
8340 reflections	Δρ_max_ = 0.18 e Å^−^^3^
491 parameters	Δρ_min_ = −0.18 e Å^−^^3^
0 restraints	

### 7-(Diethylamino)-2,2-diphenyl-1,3-dioxa-2-borata-1,2-dihydronaphthalene (I) Special details

**Table:**

Geometry. All esds (except the esd in the dihedral angle between two l.s. planes) are estimated using the full covariance matrix. The cell esds are taken into account individually in the estimation of esds in distances, angles and torsion angles; correlations between esds in cell parameters are only used when they are defined by crystal symmetry. An approximate (isotropic) treatment of cell esds is used for estimating esds involving l.s. planes.

### 7-(Diethylamino)-2,2-diphenyl-1,3-dioxa-2-borata-1,2-dihydronaphthalene (I) Fractional atomic coordinates and isotropic or equivalent isotropic displacement parameters (Å^2^)

**Table:**

	*x*	*y*	*z*	*U*_iso_*/*U*_eq_	
O1	0.53603 (19)	−0.09682 (14)	0.73035 (12)	0.0662 (5)	
N1	0.8206 (3)	0.1521 (2)	0.42080 (15)	0.0827 (8)	
C1	0.5824 (3)	−0.1290 (2)	0.66118 (19)	0.0762 (9)	
H1	0.572647	−0.203835	0.649876	0.091*	
B1	0.5754 (3)	0.0205 (2)	0.75708 (19)	0.0552 (8)	
O2	0.59385 (18)	0.09724 (13)	0.67987 (11)	0.0602 (5)	
N2	0.3505 (2)	0.6467 (2)	0.95420 (14)	0.0666 (6)	
B2	0.7059 (3)	0.5222 (2)	0.66826 (19)	0.0524 (7)	
O3	0.65253 (18)	0.40191 (14)	0.66678 (12)	0.0650 (5)	
O4	0.60878 (15)	0.59518 (13)	0.71133 (10)	0.0535 (5)	
C24	0.5782 (3)	0.3668 (2)	0.72724 (17)	0.0596 (7)	
H24	0.558192	0.290986	0.730969	0.072*	
C25	0.5264 (2)	0.4334 (2)	0.78661 (16)	0.0524 (6)	
C26	0.4490 (3)	0.3909 (2)	0.85421 (18)	0.0649 (8)	
H26	0.436344	0.313740	0.861057	0.078*	
C27	0.3929 (3)	0.4591 (3)	0.90907 (18)	0.0678 (8)	
H27	0.344752	0.428083	0.953768	0.081*	
C28	0.4068 (2)	0.5784 (2)	0.89925 (16)	0.0542 (6)	
C29	0.4804 (2)	0.6220 (2)	0.83050 (15)	0.0501 (6)	
H29	0.487968	0.699660	0.821733	0.060*	
C30	0.5411 (2)	0.5527 (2)	0.77622 (15)	0.0465 (6)	
C31	0.8321 (3)	0.5120 (2)	0.71860 (16)	0.0581 (7)	
C32	0.9044 (3)	0.4135 (3)	0.7290 (2)	0.0870 (10)	
H32	0.874740	0.346663	0.711373	0.104*	
C33	1.0206 (5)	0.4131 (5)	0.7654 (3)	0.1164 (16)	
H33	1.067531	0.346184	0.771536	0.140*	
C34	1.0660 (4)	0.5099 (6)	0.7922 (3)	0.1203 (18)	
H34	1.143984	0.509266	0.815794	0.144*	
C35	0.9976 (4)	0.6054 (4)	0.7842 (2)	0.1055 (13)	
H35	1.027674	0.671192	0.803419	0.127*	
C36	0.8826 (3)	0.6077 (3)	0.7477 (2)	0.0795 (9)	
H36	0.837399	0.675585	0.742486	0.095*	
C37	0.7232 (3)	0.5703 (2)	0.57312 (16)	0.0529 (6)	
C38	0.8380 (3)	0.5875 (3)	0.53268 (19)	0.0810 (10)	
H38	0.910560	0.571497	0.561650	0.097*	
C39	0.8491 (4)	0.6280 (3)	0.4501 (2)	0.0946 (11)	
H39	0.928081	0.637633	0.424401	0.114*	
C40	0.7446 (4)	0.6536 (3)	0.4067 (2)	0.0833 (10)	
H40	0.751450	0.681488	0.351510	0.100*	
C41	0.6304 (4)	0.6378 (3)	0.4449 (2)	0.0820 (9)	
H41	0.558312	0.655347	0.415734	0.098*	
C42	0.6199 (3)	0.5962 (2)	0.52636 (19)	0.0695 (8)	
H42	0.540374	0.585047	0.550779	0.083*	
C43	0.2803 (3)	0.6019 (3)	1.02979 (19)	0.0863 (10)	
H43A	0.284960	0.654840	1.072724	0.104*	
H43B	0.320042	0.531585	1.049803	0.104*	
C44	0.1453 (4)	0.5818 (4)	1.0156 (2)	0.1313 (17)	
H44A	0.139749	0.530015	0.972839	0.197*	
H44B	0.104013	0.651879	0.998807	0.197*	
H44C	0.105463	0.550642	1.066115	0.197*	
C45	0.3515 (3)	0.7704 (3)	0.9396 (2)	0.0747 (9)	
H45A	0.435359	0.792552	0.920821	0.090*	
H45B	0.331727	0.804384	0.991659	0.090*	
C46	0.2599 (3)	0.8153 (3)	0.8763 (3)	0.1022 (12)	
H46A	0.279444	0.782755	0.824272	0.153*	
H46B	0.265574	0.895825	0.868917	0.153*	
H46C	0.176286	0.796109	0.895283	0.153*	
C2	0.6454 (3)	−0.0609 (2)	0.60257 (18)	0.0752 (9)	
C3	0.7001 (5)	−0.1017 (3)	0.5287 (2)	0.1132 (15)	
H3	0.697501	−0.178385	0.520780	0.136*	
C4	0.7556 (4)	−0.0329 (3)	0.4695 (2)	0.1091 (14)	
H4	0.790806	−0.062657	0.421704	0.131*	
C5	0.7612 (3)	0.0854 (3)	0.47927 (18)	0.0736 (9)	
C6	0.7043 (3)	0.1275 (2)	0.55203 (16)	0.0640 (8)	
H6	0.704011	0.204556	0.558861	0.077*	
C7	0.6496 (3)	0.0568 (2)	0.61259 (16)	0.0595 (7)	
C8	0.7027 (3)	0.0022 (2)	0.80481 (17)	0.0607 (7)	
C9	0.7525 (3)	−0.1014 (3)	0.83307 (19)	0.0749 (9)	
H9	0.710055	−0.166418	0.823903	0.090*	
C10	0.8643 (4)	−0.1113 (4)	0.8748 (2)	0.0990 (12)	
H10	0.896208	−0.182034	0.892340	0.119*	
C11	0.9272 (4)	−0.0162 (5)	0.8900 (2)	0.1057 (13)	
H11	1.001230	−0.022599	0.918513	0.127*	
C12	0.8820 (4)	0.0869 (4)	0.8635 (3)	0.1111 (14)	
H12	0.925109	0.151270	0.873340	0.133*	
C13	0.7716 (3)	0.0957 (3)	0.8218 (2)	0.0878 (10)	
H13	0.741521	0.167075	0.804279	0.105*	
C14	0.4570 (2)	0.0692 (2)	0.80939 (16)	0.0516 (6)	
C15	0.4528 (3)	0.0731 (2)	0.89529 (16)	0.0577 (7)	
H15	0.523163	0.049553	0.924471	0.069*	
C16	0.3469 (3)	0.1109 (2)	0.93871 (19)	0.0686 (8)	
H16	0.346943	0.112859	0.996214	0.082*	
C17	0.2422 (3)	0.1453 (3)	0.8971 (2)	0.0777 (9)	
H17	0.170708	0.170022	0.926256	0.093*	
C18	0.2428 (3)	0.1432 (3)	0.8124 (2)	0.0801 (9)	
H18	0.171788	0.166823	0.783991	0.096*	
C19	0.3491 (3)	0.1058 (2)	0.76901 (19)	0.0669 (8)	
H19	0.348398	0.105227	0.711452	0.080*	
C20	0.8804 (4)	0.1091 (3)	0.3436 (2)	0.1040 (13)	
H20A	0.882825	0.169580	0.300228	0.125*	
H20B	0.830299	0.049568	0.325029	0.125*	
C21	1.0107 (6)	0.0647 (5)	0.3574 (3)	0.185 (3)	
H21A	1.047292	0.039731	0.306199	0.278*	
H21B	1.008222	0.002540	0.398464	0.278*	
H21C	1.060296	0.123254	0.376336	0.278*	
C22	0.8287 (4)	0.2749 (3)	0.4276 (2)	0.0998 (12)	
H22A	0.832908	0.290873	0.485517	0.120*	
H22B	0.904446	0.301953	0.398384	0.120*	
C23	0.7185 (5)	0.3336 (4)	0.3917 (4)	0.151 (2)	
H23A	0.723936	0.413135	0.397332	0.227*	
H23B	0.643585	0.306389	0.420468	0.227*	
H23C	0.715912	0.319542	0.333921	0.227*	

### 7-(Diethylamino)-2,2-diphenyl-1,3-dioxa-2-borata-1,2-dihydronaphthalene (I) Atomic displacement parameters (Å^2^)

**Table:**

	*U*^11^	*U*^22^	*U*^33^	*U*^12^	*U*^13^	*U*^23^
O1	0.0925 (15)	0.0455 (10)	0.0604 (12)	−0.0145 (9)	0.0079 (10)	−0.0031 (9)
N1	0.112 (2)	0.0756 (18)	0.0584 (15)	−0.0104 (15)	0.0241 (15)	−0.0058 (13)
C1	0.120 (3)	0.0468 (16)	0.0630 (19)	−0.0167 (17)	−0.0004 (18)	−0.0103 (14)
B1	0.063 (2)	0.0452 (16)	0.0575 (18)	−0.0090 (14)	0.0031 (15)	−0.0031 (14)
O2	0.0827 (13)	0.0440 (10)	0.0532 (11)	−0.0112 (9)	0.0139 (9)	−0.0054 (8)
N2	0.0601 (15)	0.0826 (18)	0.0561 (14)	−0.0031 (12)	0.0089 (12)	−0.0027 (12)
B2	0.0564 (18)	0.0409 (15)	0.0593 (18)	0.0003 (13)	0.0050 (15)	−0.0045 (13)
O3	0.0817 (14)	0.0476 (10)	0.0655 (12)	−0.0067 (9)	0.0066 (11)	−0.0067 (9)
O4	0.0563 (11)	0.0435 (9)	0.0586 (11)	−0.0032 (8)	0.0111 (9)	0.0036 (8)
C24	0.0701 (19)	0.0446 (15)	0.0642 (18)	−0.0079 (13)	−0.0086 (15)	0.0028 (13)
C25	0.0535 (15)	0.0445 (14)	0.0585 (16)	−0.0050 (12)	−0.0050 (13)	0.0045 (12)
C26	0.0685 (19)	0.0525 (16)	0.0717 (19)	−0.0119 (14)	0.0004 (15)	0.0133 (14)
C27	0.0657 (19)	0.0707 (19)	0.0639 (18)	−0.0080 (15)	0.0100 (15)	0.0143 (15)
C28	0.0471 (15)	0.0621 (17)	0.0527 (15)	−0.0036 (12)	−0.0014 (12)	0.0028 (13)
C29	0.0499 (15)	0.0432 (13)	0.0563 (15)	−0.0023 (11)	0.0017 (12)	0.0016 (12)
C30	0.0408 (13)	0.0469 (14)	0.0514 (14)	−0.0048 (11)	−0.0041 (11)	0.0038 (11)
C31	0.0614 (17)	0.0638 (17)	0.0472 (15)	0.0045 (14)	0.0063 (13)	0.0036 (13)
C32	0.092 (3)	0.092 (2)	0.074 (2)	0.035 (2)	0.0040 (19)	−0.0008 (18)
C33	0.098 (3)	0.158 (5)	0.087 (3)	0.067 (3)	0.001 (2)	0.009 (3)
C34	0.064 (3)	0.226 (6)	0.068 (3)	0.012 (3)	0.0014 (19)	0.009 (3)
C35	0.081 (3)	0.150 (4)	0.087 (3)	−0.032 (3)	−0.014 (2)	0.002 (3)
C36	0.075 (2)	0.083 (2)	0.081 (2)	−0.0115 (18)	−0.0165 (18)	0.0019 (18)
C37	0.0593 (17)	0.0441 (14)	0.0552 (15)	−0.0008 (12)	−0.0014 (13)	−0.0037 (11)
C38	0.068 (2)	0.107 (3)	0.0634 (19)	0.0043 (18)	0.0069 (16)	0.0151 (17)
C39	0.090 (3)	0.120 (3)	0.068 (2)	0.003 (2)	0.019 (2)	0.016 (2)
C40	0.121 (3)	0.075 (2)	0.0520 (18)	−0.001 (2)	0.000 (2)	0.0031 (15)
C41	0.097 (3)	0.086 (2)	0.065 (2)	−0.0049 (19)	−0.0242 (19)	−0.0045 (17)
C42	0.070 (2)	0.075 (2)	0.0646 (19)	−0.0096 (15)	−0.0085 (16)	−0.0056 (15)
C43	0.085 (2)	0.119 (3)	0.0552 (18)	−0.007 (2)	0.0077 (17)	−0.0071 (18)
C44	0.081 (3)	0.222 (5)	0.089 (3)	−0.042 (3)	0.018 (2)	0.004 (3)
C45	0.067 (2)	0.075 (2)	0.084 (2)	−0.0118 (16)	0.0163 (17)	−0.0291 (17)
C46	0.075 (2)	0.070 (2)	0.161 (4)	0.0040 (18)	−0.010 (2)	0.001 (2)
C2	0.124 (3)	0.0470 (16)	0.0546 (17)	−0.0147 (16)	0.0106 (17)	−0.0072 (13)
C3	0.218 (5)	0.0530 (19)	0.068 (2)	−0.019 (2)	0.026 (3)	−0.0160 (17)
C4	0.201 (4)	0.066 (2)	0.058 (2)	−0.003 (2)	0.031 (2)	−0.0157 (17)
C5	0.104 (2)	0.0641 (19)	0.0519 (17)	−0.0083 (17)	0.0107 (16)	−0.0035 (14)
C6	0.088 (2)	0.0499 (15)	0.0541 (16)	−0.0097 (14)	0.0108 (15)	−0.0047 (13)
C7	0.080 (2)	0.0506 (16)	0.0479 (15)	−0.0087 (14)	0.0052 (14)	−0.0071 (12)
C8	0.0573 (17)	0.0616 (17)	0.0621 (17)	0.0040 (14)	0.0142 (14)	−0.0098 (14)
C9	0.072 (2)	0.082 (2)	0.068 (2)	0.0098 (17)	0.0097 (17)	0.0014 (16)
C10	0.084 (3)	0.122 (3)	0.086 (3)	0.032 (2)	0.010 (2)	0.009 (2)
C11	0.065 (2)	0.161 (4)	0.092 (3)	0.022 (3)	−0.004 (2)	−0.025 (3)
C12	0.063 (2)	0.129 (4)	0.146 (4)	0.005 (2)	−0.011 (2)	−0.046 (3)
C13	0.065 (2)	0.084 (2)	0.118 (3)	0.0039 (18)	−0.010 (2)	−0.026 (2)
C14	0.0516 (15)	0.0459 (14)	0.0561 (16)	−0.0074 (11)	0.0000 (12)	0.0067 (11)
C15	0.0536 (16)	0.0622 (17)	0.0550 (16)	0.0028 (13)	0.0038 (13)	0.0078 (13)
C16	0.068 (2)	0.0730 (19)	0.0616 (18)	0.0023 (15)	0.0165 (15)	0.0040 (15)
C17	0.058 (2)	0.073 (2)	0.097 (3)	0.0061 (15)	0.0165 (18)	0.0113 (18)
C18	0.0563 (19)	0.080 (2)	0.102 (3)	0.0042 (16)	−0.0100 (18)	0.0170 (19)
C19	0.0656 (19)	0.0669 (18)	0.0668 (18)	−0.0041 (15)	−0.0071 (15)	0.0095 (14)
C20	0.152 (4)	0.102 (3)	0.055 (2)	−0.004 (3)	0.021 (2)	−0.0012 (18)
C21	0.173 (5)	0.236 (7)	0.130 (4)	0.085 (5)	0.069 (4)	0.022 (4)
C22	0.117 (3)	0.100 (3)	0.081 (2)	−0.040 (2)	0.033 (2)	−0.008 (2)
C23	0.154 (5)	0.105 (4)	0.189 (5)	0.016 (3)	0.040 (4)	−0.002 (3)

### 7-(Diethylamino)-2,2-diphenyl-1,3-dioxa-2-borata-1,2-dihydronaphthalene (I) Geometric parameters (Å, º)

**Table:**

O1—C1	1.279 (3)	C43—H43B	0.9700
O1—B1	1.562 (3)	C43—C44	1.497 (5)
N1—C5	1.341 (4)	C44—H44A	0.9600
N1—C20	1.490 (4)	C44—H44B	0.9600
N1—C22	1.477 (4)	C44—H44C	0.9600
C1—H1	0.9300	C45—H45A	0.9700
C1—C2	1.370 (4)	C45—H45B	0.9700
B1—O2	1.504 (3)	C45—C46	1.507 (5)
B1—C8	1.593 (4)	C46—H46A	0.9600
B1—C14	1.606 (4)	C46—H46B	0.9600
O2—C7	1.328 (3)	C46—H46C	0.9600
N2—C28	1.353 (3)	C2—C3	1.414 (4)
N2—C43	1.481 (4)	C2—C7	1.422 (4)
N2—C45	1.474 (4)	C3—H3	0.9300
B2—O3	1.557 (3)	C3—C4	1.345 (5)
B2—O4	1.510 (3)	C4—H4	0.9300
B2—C31	1.600 (4)	C4—C5	1.430 (4)
B2—C37	1.608 (4)	C5—C6	1.414 (4)
O3—C24	1.286 (3)	C6—H6	0.9300
O4—C30	1.327 (3)	C6—C7	1.369 (3)
C24—H24	0.9300	C8—C9	1.386 (4)
C24—C25	1.372 (4)	C8—C13	1.398 (4)
C25—C26	1.415 (4)	C9—H9	0.9300
C25—C30	1.430 (3)	C9—C10	1.394 (5)
C26—H26	0.9300	C10—H10	0.9300
C26—C27	1.350 (4)	C10—C11	1.372 (6)
C27—H27	0.9300	C11—H11	0.9300
C27—C28	1.428 (4)	C11—C12	1.354 (6)
C28—C29	1.411 (3)	C12—H12	0.9300
C29—H29	0.9300	C12—C13	1.380 (5)
C29—C30	1.373 (3)	C13—H13	0.9300
C31—C32	1.389 (4)	C14—C15	1.389 (4)
C31—C36	1.390 (4)	C14—C19	1.392 (4)
C32—H32	0.9300	C15—H15	0.9300
C32—C33	1.395 (6)	C15—C16	1.385 (4)
C33—H33	0.9300	C16—H16	0.9300
C33—C34	1.364 (6)	C16—C17	1.367 (4)
C34—H34	0.9300	C17—H17	0.9300
C34—C35	1.336 (6)	C17—C18	1.368 (4)
C35—H35	0.9300	C18—H18	0.9300
C35—C36	1.383 (5)	C18—C19	1.386 (4)
C36—H36	0.9300	C19—H19	0.9300
C37—C38	1.378 (4)	C20—H20A	0.9700
C37—C42	1.378 (4)	C20—H20B	0.9700
C38—H38	0.9300	C20—C21	1.494 (6)
C38—C39	1.389 (4)	C21—H21A	0.9600
C39—H39	0.9300	C21—H21B	0.9600
C39—C40	1.358 (5)	C21—H21C	0.9600
C40—H40	0.9300	C22—H22A	0.9700
C40—C41	1.355 (5)	C22—H22B	0.9700
C41—H41	0.9300	C22—C23	1.471 (6)
C41—C42	1.376 (4)	C23—H23A	0.9600
C42—H42	0.9300	C23—H23B	0.9600
C43—H43A	0.9700	C23—H23C	0.9600
			
C1—O1—B1	117.3 (2)	H44A—C44—H44C	109.5
C5—N1—C20	123.0 (3)	H44B—C44—H44C	109.5
C5—N1—C22	122.1 (3)	N2—C45—H45A	108.9
C22—N1—C20	114.9 (3)	N2—C45—H45B	108.9
O1—C1—H1	117.7	N2—C45—C46	113.3 (3)
O1—C1—C2	124.6 (3)	H45A—C45—H45B	107.7
C2—C1—H1	117.7	C46—C45—H45A	108.9
O1—B1—C8	107.7 (2)	C46—C45—H45B	108.9
O1—B1—C14	105.8 (2)	C45—C46—H46A	109.5
O2—B1—O1	108.1 (2)	C45—C46—H46B	109.5
O2—B1—C8	111.0 (2)	C45—C46—H46C	109.5
O2—B1—C14	107.4 (2)	H46A—C46—H46B	109.5
C8—B1—C14	116.4 (2)	H46A—C46—H46C	109.5
C7—O2—B1	119.3 (2)	H46B—C46—H46C	109.5
C28—N2—C43	122.1 (3)	C1—C2—C3	122.5 (3)
C28—N2—C45	121.3 (2)	C1—C2—C7	119.6 (3)
C45—N2—C43	116.5 (2)	C3—C2—C7	117.8 (3)
O3—B2—C31	107.8 (2)	C2—C3—H3	119.1
O3—B2—C37	106.8 (2)	C4—C3—C2	121.9 (3)
O4—B2—O3	107.9 (2)	C4—C3—H3	119.1
O4—B2—C31	110.8 (2)	C3—C4—H4	119.6
O4—B2—C37	108.3 (2)	C3—C4—C5	120.7 (3)
C31—B2—C37	115.0 (2)	C5—C4—H4	119.6
C24—O3—B2	118.3 (2)	N1—C5—C4	119.8 (3)
C30—O4—B2	120.12 (19)	N1—C5—C6	122.3 (3)
O3—C24—H24	117.7	C6—C5—C4	117.8 (3)
O3—C24—C25	124.6 (2)	C5—C6—H6	119.4
C25—C24—H24	117.7	C7—C6—C5	121.1 (3)
C24—C25—C26	123.0 (2)	C7—C6—H6	119.4
C24—C25—C30	118.9 (2)	O2—C7—C2	118.6 (2)
C26—C25—C30	117.8 (2)	O2—C7—C6	120.8 (2)
C25—C26—H26	119.0	C6—C7—C2	120.6 (2)
C27—C26—C25	121.9 (3)	C9—C8—B1	125.0 (3)
C27—C26—H26	119.0	C9—C8—C13	115.4 (3)
C26—C27—H27	119.6	C13—C8—B1	119.6 (3)
C26—C27—C28	120.8 (2)	C8—C9—H9	119.0
C28—C27—H27	119.6	C8—C9—C10	122.0 (3)
N2—C28—C27	120.8 (2)	C10—C9—H9	119.0
N2—C28—C29	121.4 (2)	C9—C10—H10	120.1
C29—C28—C27	117.8 (2)	C11—C10—C9	119.8 (4)
C28—C29—H29	119.2	C11—C10—H10	120.1
C30—C29—C28	121.6 (2)	C10—C11—H11	119.9
C30—C29—H29	119.2	C12—C11—C10	120.3 (4)
O4—C30—C25	119.0 (2)	C12—C11—H11	119.9
O4—C30—C29	120.8 (2)	C11—C12—H12	120.3
C29—C30—C25	120.0 (2)	C11—C12—C13	119.5 (4)
C32—C31—B2	124.0 (3)	C13—C12—H12	120.3
C32—C31—C36	115.6 (3)	C8—C13—H13	118.5
C36—C31—B2	120.0 (3)	C12—C13—C8	123.0 (4)
C31—C32—H32	119.4	C12—C13—H13	118.5
C31—C32—C33	121.2 (4)	C15—C14—B1	123.5 (2)
C33—C32—H32	119.4	C15—C14—C19	116.5 (2)
C32—C33—H33	119.7	C19—C14—B1	119.9 (2)
C34—C33—C32	120.6 (4)	C14—C15—H15	119.0
C34—C33—H33	119.7	C16—C15—C14	121.9 (3)
C33—C34—H34	120.2	C16—C15—H15	119.0
C35—C34—C33	119.5 (5)	C15—C16—H16	120.0
C35—C34—H34	120.2	C17—C16—C15	120.0 (3)
C34—C35—H35	119.7	C17—C16—H16	120.0
C34—C35—C36	120.7 (4)	C16—C17—H17	120.1
C36—C35—H35	119.7	C16—C17—C18	119.8 (3)
C31—C36—H36	118.8	C18—C17—H17	120.1
C35—C36—C31	122.3 (4)	C17—C18—H18	120.0
C35—C36—H36	118.8	C17—C18—C19	120.1 (3)
C38—C37—B2	123.9 (3)	C19—C18—H18	120.0
C38—C37—C42	115.7 (3)	C14—C19—H19	119.2
C42—C37—B2	120.4 (2)	C18—C19—C14	121.7 (3)
C37—C38—H38	118.9	C18—C19—H19	119.2
C37—C38—C39	122.2 (3)	N1—C20—H20A	109.3
C39—C38—H38	118.9	N1—C20—H20B	109.3
C38—C39—H39	120.0	N1—C20—C21	111.6 (3)
C40—C39—C38	120.1 (3)	H20A—C20—H20B	108.0
C40—C39—H39	120.0	C21—C20—H20A	109.3
C39—C40—H40	120.5	C21—C20—H20B	109.3
C41—C40—C39	119.1 (3)	C20—C21—H21A	109.5
C41—C40—H40	120.5	C20—C21—H21B	109.5
C40—C41—H41	119.7	C20—C21—H21C	109.5
C40—C41—C42	120.6 (3)	H21A—C21—H21B	109.5
C42—C41—H41	119.7	H21A—C21—H21C	109.5
C37—C42—H42	118.8	H21B—C21—H21C	109.5
C41—C42—C37	122.4 (3)	N1—C22—H22A	109.6
C41—C42—H42	118.8	N1—C22—H22B	109.6
N2—C43—H43A	108.9	H22A—C22—H22B	108.1
N2—C43—H43B	108.9	C23—C22—N1	110.2 (4)
N2—C43—C44	113.3 (3)	C23—C22—H22A	109.6
H43A—C43—H43B	107.7	C23—C22—H22B	109.6
C44—C43—H43A	108.9	C22—C23—H23A	109.5
C44—C43—H43B	108.9	C22—C23—H23B	109.5
C43—C44—H44A	109.5	C22—C23—H23C	109.5
C43—C44—H44B	109.5	H23A—C23—H23B	109.5
C43—C44—H44C	109.5	H23A—C23—H23C	109.5
H44A—C44—H44B	109.5	H23B—C23—H23C	109.5
			
O1—C1—C2—C3	177.0 (4)	C31—B2—O4—C30	−79.9 (3)
O1—C1—C2—C7	−7.5 (5)	C31—B2—C37—C38	8.7 (4)
O1—B1—O2—C7	−40.4 (3)	C31—B2—C37—C42	−172.0 (2)
O1—B1—C8—C9	−13.0 (4)	C31—C32—C33—C34	0.3 (6)
O1—B1—C8—C13	167.9 (2)	C32—C31—C36—C35	0.4 (4)
O1—B1—C14—C15	105.6 (3)	C32—C33—C34—C35	0.8 (7)
O1—B1—C14—C19	−71.5 (3)	C33—C34—C35—C36	−1.3 (6)
N1—C5—C6—C7	−176.8 (3)	C34—C35—C36—C31	0.7 (5)
C1—O1—B1—O2	33.7 (3)	C36—C31—C32—C33	−0.9 (4)
C1—O1—B1—C8	−86.4 (3)	C37—B2—O3—C24	−147.9 (2)
C1—O1—B1—C14	148.5 (3)	C37—B2—O4—C30	153.1 (2)
C1—C2—C3—C4	176.2 (4)	C37—B2—C31—C32	−96.0 (3)
C1—C2—C7—O2	1.3 (5)	C37—B2—C31—C36	77.3 (3)
C1—C2—C7—C6	−175.3 (3)	C37—C38—C39—C40	0.9 (6)
B1—O1—C1—C2	−12.1 (5)	C38—C37—C42—C41	−0.8 (4)
B1—O2—C7—C2	24.8 (4)	C38—C39—C40—C41	−0.7 (6)
B1—O2—C7—C6	−158.6 (3)	C39—C40—C41—C42	−0.2 (5)
B1—C8—C9—C10	−179.7 (3)	C40—C41—C42—C37	1.0 (5)
B1—C8—C13—C12	179.4 (3)	C42—C37—C38—C39	−0.2 (5)
B1—C14—C15—C16	−177.0 (2)	C43—N2—C28—C27	4.5 (4)
B1—C14—C19—C18	176.8 (3)	C43—N2—C28—C29	−175.7 (2)
O2—B1—C8—C9	−131.1 (3)	C43—N2—C45—C46	−100.7 (3)
O2—B1—C8—C13	49.8 (3)	C45—N2—C28—C27	−172.7 (3)
O2—B1—C14—C15	−139.1 (2)	C45—N2—C28—C29	7.1 (4)
O2—B1—C14—C19	43.8 (3)	C45—N2—C43—C44	89.6 (4)
N2—C28—C29—C30	177.7 (2)	C2—C3—C4—C5	−0.2 (7)
B2—O3—C24—C25	10.8 (4)	C3—C2—C7—O2	177.0 (3)
B2—O4—C30—C25	−22.6 (3)	C3—C2—C7—C6	0.4 (5)
B2—O4—C30—C29	160.8 (2)	C3—C4—C5—N1	177.8 (4)
B2—C31—C32—C33	172.7 (3)	C3—C4—C5—C6	−1.3 (6)
B2—C31—C36—C35	−173.4 (3)	C4—C5—C6—C7	2.3 (5)
B2—C37—C38—C39	179.2 (3)	C5—N1—C20—C21	−85.1 (5)
B2—C37—C42—C41	179.8 (3)	C5—N1—C22—C23	−87.7 (4)
O3—B2—O4—C30	37.9 (3)	C5—C6—C7—O2	−178.4 (3)
O3—B2—C31—C32	23.0 (3)	C5—C6—C7—C2	−1.9 (5)
O3—B2—C31—C36	−163.8 (2)	C7—C2—C3—C4	0.6 (6)
O3—B2—C37—C38	−110.8 (3)	C8—B1—O2—C7	77.5 (3)
O3—B2—C37—C42	68.5 (3)	C8—B1—C14—C15	−13.9 (4)
O3—C24—C25—C26	−177.4 (3)	C8—B1—C14—C19	168.9 (2)
O3—C24—C25—C30	8.6 (4)	C8—C9—C10—C11	1.0 (5)
O4—B2—O3—C24	−31.6 (3)	C9—C8—C13—C12	0.3 (5)
O4—B2—C31—C32	140.8 (3)	C9—C10—C11—C12	−0.9 (6)
O4—B2—C31—C36	−45.9 (3)	C10—C11—C12—C13	0.6 (6)
O4—B2—C37—C38	133.2 (3)	C11—C12—C13—C8	−0.3 (6)
O4—B2—C37—C42	−47.5 (3)	C13—C8—C9—C10	−0.6 (4)
C24—C25—C26—C27	−175.9 (3)	C14—B1—O2—C7	−154.2 (2)
C24—C25—C30—O4	−2.8 (4)	C14—B1—C8—C9	105.5 (3)
C24—C25—C30—C29	173.9 (2)	C14—B1—C8—C13	−73.5 (3)
C25—C26—C27—C28	2.0 (5)	C14—C15—C16—C17	0.3 (4)
C26—C25—C30—O4	−177.1 (2)	C15—C14—C19—C18	−0.6 (4)
C26—C25—C30—C29	−0.5 (4)	C15—C16—C17—C18	−0.6 (5)
C26—C27—C28—N2	180.0 (3)	C16—C17—C18—C19	0.3 (5)
C26—C27—C28—C29	0.1 (4)	C17—C18—C19—C14	0.3 (5)
C27—C28—C29—C30	−2.5 (4)	C19—C14—C15—C16	0.2 (4)
C28—N2—C43—C44	−87.7 (4)	C20—N1—C5—C4	1.3 (5)
C28—N2—C45—C46	76.7 (3)	C20—N1—C5—C6	−179.7 (3)
C28—C29—C30—O4	179.2 (2)	C20—N1—C22—C23	90.9 (4)
C28—C29—C30—C25	2.6 (4)	C22—N1—C5—C4	179.7 (4)
C30—C25—C26—C27	−1.8 (4)	C22—N1—C5—C6	−1.2 (5)
C31—B2—O3—C24	88.0 (3)	C22—N1—C20—C21	96.3 (4)

### 7-(Diethylamino)-2,2-diphenyl-1,3-dioxa-2-borata-1,2-dihydronaphthalene (I) Hydrogen-bond geometry (Å, º)

*Cg*3, *Cg*4, *Cg*7, *Cg*8 and *Cg*9 are the centroids of rings C3–C13, C14–C19, C25–C30, C31–C36 and C37–C42, respectively.

**Table:**

*D*—H···*A*	*D*—H	H···*A*	*D*···*A*	*D*—H···*A*
C24—H24···O2	0.93	2.51	3.343 (3)	149
C1—H1···O4^i^	0.93	2.55	3.334 (3)	142
C3—H3···*Cg*9^i^	0.93	2.59	3.510 (4)	169
C23—H23*A*···*Cg*9	0.96	2.88	3.771 (5)	155
C26—H26···*Cg*4	0.93	2.66	3.562 (3)	164
C46—H46*B*···*Cg*4^ii^	0.96	2.69	3.623 (4)	164
C21—H21*A*···*Cg*3^iii^	0.96	2.87	3.815 (5)	168
C43—H43*B*···*Cg*7^iv^	0.96	2.93	3.626 (3)	129
C44—H44*C*···*Cg*8^iv^	0.96	2.95	3.876 (4)	162

Symmetry codes: (i) *x*, *y*−1, *z*; (ii) *x*, *y*+1, *z*; (iii) −*x*+2, −*y*, −*z*+1; (iv) −*x*+1, −*y*+1, −*z*+2.
