# Supplementary figures and images for: Unusual reaction of (E)-2-[(benzo[d]thia­zol-2-yl­imino)­meth­yl]-5-(di­ethyl­amino)­phenol with tri­phenyl­borane: crystal structures and optical properties
Source: Acta Crystallogr E Crystallogr Commun. 2023 Oct 3;79(Pt 11):982–7. doi: 10.1107/S2056989023008514 (PMC10626958; doi:10.1107/S2056989023008514)

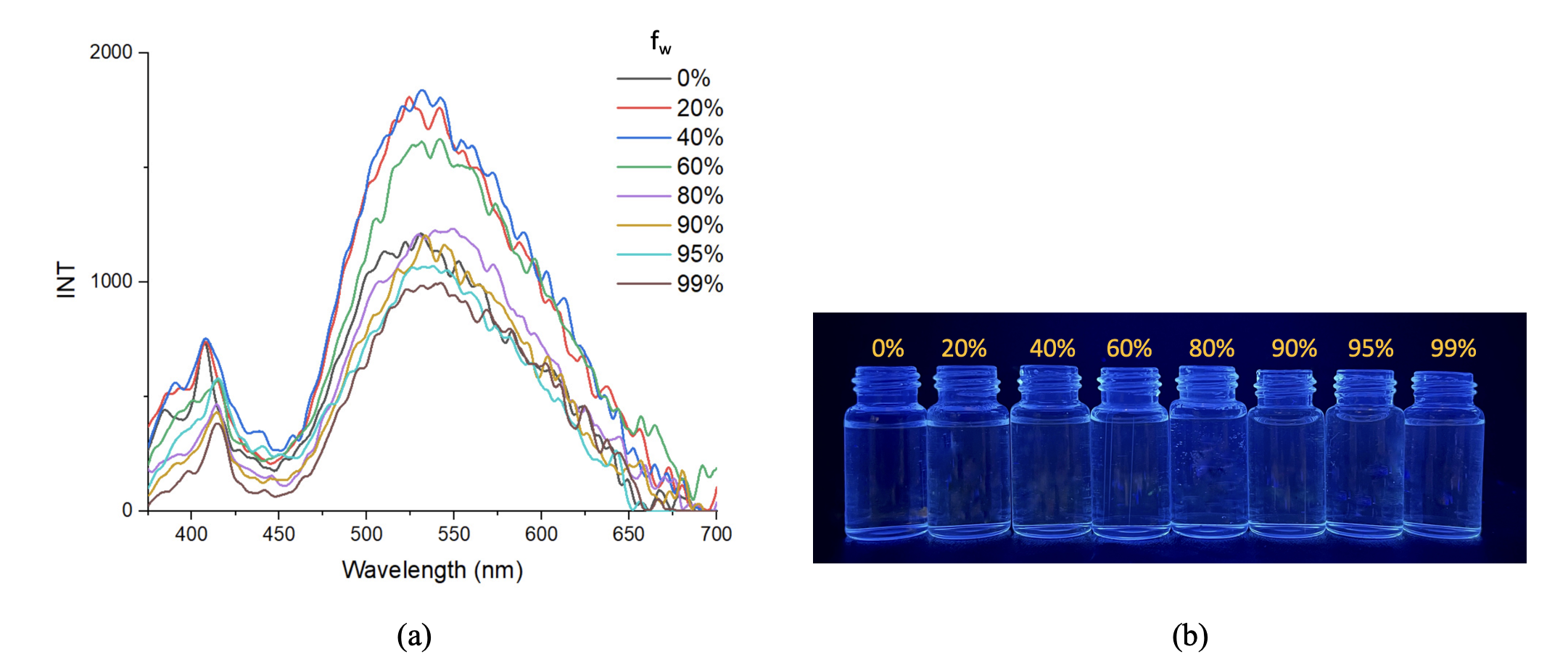

Supplement: Supplementary file 5 [file e-79-00982-sup5.png]
